# Electric vehicle integrated tidal-solar-wind-hydro-thermal systems for strengthing the microgrid and environment sustainability

**DOI:** 10.1038/s41598-025-98594-9

**Published:** 2025-04-28

**Authors:** Sunanda Hazra, Dipanjan Datta, Chandan Paul, Provas Kumar Roy, Sneha Sultana, Sajjan Kumar, Soham Dutta

**Affiliations:** 1 Department of Electrical Engineering, Haldia Institute of Technology, Haldia, India; 2 ICA Edu Skills Pvt. Ltd, Kolkata, India; 3 Department of Electrical Engineering, Dr. B. C. Roy Engineering College, Durgapur, India; 4 Department of Electrical Engineering, Kalyani Government Engineering College, Kalyani, West Bengal India; 5 Department of Electrical and Electronics Engineering, Sri Sivasubramaniya Nadar College of Engineering, Chennai, India; 6 Department of Electrical and Electronics Engineering, Manipal Institute of Technology, Manipal Academy of Higher Education, Manipal, Karnataka 576104 India

**Keywords:** Tidal, Wind, Solar, Electric vehicles (EVs), Hydro-thermal scheduling (HTS), Advanced optimization technique, Energy science and technology, Mathematics and computing

## Abstract

Incorporating electric vehicles (EVs) into the power grid significantly impacts its safe and reliable operation, while the unpredictable nature of wind power adds further complications. Solar power, though less efficient in converting sunlight to electricity compared to wind power, remains a popular renewable energy source. Combining wind and solar energy is advantageous because wind energy can be harnessed both day and night, unlike solar energy. Tidal energy also offers a reliable renewable option, although it has its own set of challenges. Consequently, the utilization of renewable energy sources (RESs) have become increasingly complex. Fossil fuels, on the other hand, are a major cause of severe pollution. This study addresses integration of wind, solar, tidal, and electric vehicles, using a unique moth-flame optimization technique, to solve the challenge of hydrothermal scheduling (HTS). The primary objective is to reduce power generation costs while adhering to various limitations, including transmission losses, thermal unit valve point effects, and RESs variability. In order to maximize energy management, several EVs are currently being built as virtual power plants (VPPs), utilizing sustainable energy sources. So, VPPs and combined renewable energy sources make the micro-grid more rigid. The objective is to minimize fuel expenditures by balancing load demand and transmission losses while satisfying all conditions. By evaluating the generation costs with MFO, this study demonstrates the effectiveness of the method and compares it with other advanced optimization techniques, highlighting its superior efficiency, utility and reliability. When the performance of normal HTS system, RES and EV based HTS system are observed, it is clearly observed that RESs based system has improved the results by 5.49% as compared to the conventional system using the suggested COMFO approach. The findings also show that EVs can effectively contribute to a hydro-thermal scheduling system with integrated renewable energy by using grid power.

## Introduction

To achieve sustainable development, developing countries must have access to clean energy. An Energy transition is necessary to conserve natural fuels, reduce greenhouse gas concentrations, and halt rising sea levels. With the daily depletion of fossil fuel sources, integrating renewable energy sources (RESs) with thermal units is essential. However, maintaining economic viability, reliability, and security in the face of intermittent nature of wind and solar power requires effective policies and dispatch methods. Fossil fuel-based energy production reduces the efficiency of thermal generating units to 50–60% and harms the environment through emissions. Thermal power plants may have an effect on the performance of grid-connected wind power generation. Controlling these plants directly impacts pollutant discharge, contributing to environmental pollution. Maximizing thermal power output can achieve both economic and environmental goals. Hydro-generation, using fewer expensive and scarce fossil fuels, lowers the environmental damage associated with thermal, diesel, and nuclear power generation. Thus, environmental degradation and fossil fuel issues significantly drive electric vehicle adoption. The use of photovoltaic (PV)-powered electric vehicles (EVs) has been implemented to reduce greenhouse gas emissions from 47 to 78%. Satisfying load demands within a designated timeframe while adhering to constraints on thermal, hydraulic, solar, wind, and electric vehicle (EV) systems is a challenging endeavor. The objective of the wind-solar-EVs integrating hydro-thermal generation scheduling (WSEHTGS) problem is to identify the optimal combinations of thermal and hydro generation. Due to the presence of a nonlinear objective function alongside both nonlinear and linear constraints, the optimal scheduling of WSEHTGS is considerably more complex than that of a simple thermal system. WSEHTGS is vital for stable grid operation, optimizing hourly water discharge, thermal power, wind, and solar generation. The problem involves inequality constraints such as electricity production, balance of power, reservoir water levels, water flow, and thermal power constraints due to restricted operating zones (ROZ). Balancing power generation and demand while meeting all constraints makes WSEHTGS nonlinear.

Various optimization strategies have been evaluated to address these challenges. Traditional optimization approaches have strong convergence but struggle with local optima. Solving differential functions was once feasible with standard optimization, but WSEHTGS’s nonlinearity complicates this. Several methods, including mathematical decomposition (MD)^1^, network flow technique (NFT)^2^, branch-and-bound algorithm^3^, and Lagrangian relaxation (LR)^4^, require more time and iterations, increasing memory size to find optimal solutions. Evolutionary algorithms have been developed to overcome traditional optimization shortcomings. For example, Hazra et al.^5^ developed combined economic emission dispatch for wind-based systems. Improved Borg algorithm^6^ minimized costs and emissions in a wind-based hydro-thermal scheduling problem^7,8^. Combining HTS with wind and solar power aims to maximize renewable resource power^9^. Statistical process control monitored and controlled the system. Probability production simulation and chaotic self-adaptive differential mutation operator in harmony search algorithm were also proposed. Niu et al.^10^ used pitch adjustment to deliver disruptive information and harmony memory for economic dispatch problems. Quantum-behaved particle swarm optimization (MOQPSO)^11^ and genetic algorithm based on non-dominated sorting (NSGA)^12^ addressed multi-objective HTS problems. Hemmati et al.^13^ considered uncertainties like wind speed, water inflows, and power demand. Li et al.^14^ included ramp rate functions in HTS models for efficient power generation with lower emissions. Hydro unit performance curves were modeled using general formulations^15–17^. Modified approaches have been enhanced using search ability with different constraints^18^. Queiroz^19^ scheduled future water availability in complex HTS systems. Elkadeem et al.^20^ evaluated power micro-grid sustainability. Various meta-heuristic algorithms^21^, including PSO, slime mould algorithm (SMA)^22^, and the moth-flame optimization algorithm (MFOA)^23^, minimized generation cost and pollution^24^. Hazra et al. introduced an oppositional grasshopper algorithm^25^ for integration of wind power. For hydro-thermal scheduling challenges, Yin et al.^26^ deployed crisscross optimization. Roy et al.^27^ reduced generation costs by integrating renewable energy. Wu et al.^28^ used meta-heuristics for single objective problems. Approaches to handle wind and PV power uncertainties were proposed^29,30^. Mustafa İnci and colleagues^31^ proposed a method to enhance the optimization of electric vehicle (EV) charging and discharging processes. Meanwhile, Muhammad Mohsin Ansari and his team^32^ applied the point estimate method (PEM) to address uncertainties associated with wind and solar power in hydro-thermal systems (HTS). Nonetheless, the whale optimization algorithm (WOA)^33^ is known for its slow convergence and tendency to converge prematurely. The study suggested^34^ using a time-varying mutation scale (TVMS) in conjunction with fast convergence evolutionary programming (FCEP) to address the issue of hydrothermal scheduling in a grid that includes pumped-storage-hydraulic units and renewable energy sources. Demand-side management, a technique for monitoring and regulating the demand for power in real-time to maintain stability, is also incorporated into the system. In order to handle the intermittent nature of renewable energy sources, the manuscript^35^ involved quasi-oppositional fast convergence real-coded genetic algorithm (QOFC-RCGA) to optimize the generation schedule of a system containing pumped-storage hydro plants (PSHPs). PSHPs provide system flexibility by storing energy during periods of high generation or low demand and releasing it during peak periods. To improve scheduling efficiency, the system takes renewable energy and load demand volatility into account. In^36^ authors used modified artificial hummingbird algorithm (MAHA) that makes use of Levy flying and pitch adjustment actions. Four short term hydrothermal scheduling (STHS) examples are used to test MAHA, including uncertainty modeling with lognormal and Weibull distributions and integration of renewable energy. Its effectiveness is verified using AHA and other cutting-edge techniques.In order to lower generation costs, article^37^ proposed an ideal day-ahead scheduling model for wind, solar, and hydrothermal systems with pumped storage plants (PSPs). The short-term scheduling problem is solved using an improved cheetah optimizer (ICO), which has taken renewable uncertainties into account in addition to thermal, hydraulic, and network restrictions. The fuel costs, emissions, convergence rate, and calculation time of the suggested ICO method are compared to those of comparable algorithms. The outcomes validate its efficacy in optimizing OWSPHTS in the real world. In order to overcome drawbacks such as stagnation and poor diversity in complicated optimization problems, the improved quadratic interpolation optimization (IQIO) in^38^ has improved on the original QIO by adding Weibull flight motion, chaotic mutation, and PDO processes. IQIO efficiently handles restrictions and enhances solution quality when used for short-term hydrothermal scheduling (STHS) with system uncertainties and renewable energy integration. Using a self-adaptive crystal structure algorithm (SACRYSTAL)^39^, this work presented a unique energy management strategy (EMS) for microgrids that aim to integrate plug-in hybrid electric vehicles (PHEVs) with renewable energy sources (RESs). The connecting of various energy supply subsystems to meet a range of user needs and improve operating efficiency is the main focus of this paper’s investigation^40^ of regional integrated energy systems (RIES). Using the multi-objective chaotic artificial hummingbird algorithm, a new low-carbon economic dispatch technique is presented. The shortcomings of certain classically based methods, such as the LR^41^, LP^42^, NP^43^, QP^44^ have been illustrated by the authors. Many evolutionary algorithms fall short of providing optimal solutions for nonlinear problems. To account for the influence of virtual power plants (VPPs), the authors introduced moth flame optimization (MFO) for integrating EVs with wind, solar, and tidal energy in hydro-thermal systems. The merits and demerits of the existing algorithms are listed in Table 1.

**Table 1:** Literature review of the existing algorithms.

Name	Type	Mechanisms	Merits	Demerits
LR^4,41^	Classical method	Iterative optimization	Many ways to obtain feasibility	Computationally expensive, sensitive in choice of control parameters
MIP^17^		Handles linear objective function where at least one variable is integer	Optimization process is fast and better accuracy	Impossible to take nonlinear effects
LP^42^		Mathematical model	Better resource allocation, streamlined decision-making	Assumption on linearity, errors sensitivity
NP^43^		Objective functions are non-linear based	Better flexibility and accuracy	More memory required
QP^44^		Nonlinear programming	Simple for equality constraints problem	More simulation time and complex
PSO^21^	Evolutionary algorithm	Based of behabiour of swarm of birds	CEasy implementation	convergerges to suboptimal solutions
WOA^33^		Encircling prey, Bubble-net attacking technique of humpback whales	Can overcome local optima	Slow convergence speed
CRO^27^		Chemical reaction based	Greater flexibility	Not applicable for large scale problem
TLBO^45^		Population based, teaching-learning	Less parameters required	Poor population diversity
MFO^7,46^		Transverse orientation	Fast converging	Sensitive in initial population
CWOA^47^		Based on encirclement prey and bubble net searching	Can overcome local optima	Better convergence speed than WOA.
QOWOA^47^		Encirclement prey and bubble net searching is used	Can overcome local optima	Superior convergence profile compared to WOA.
CQOWOA^47^		Based on chattic quasioppositional concept, encirclement prey and bubble net searching	Avoid local optimality	Better convergence characteristic than WOA and QOWOA.
QOCRO^5^		Quasioppositional and Chemical reaction concept is used optimization	needs more memory	Slow convergence proficiency
QPSO^11^		Needs more memory	Better convergence speed	Better convergence speed
CSMA^22^		Based on Slime molds’ behavior, morphological changes in foraging and chaotic concept.	Can overcome local optimality	FAST convergence
HSA^24^		Harmony Search is a metaheuristic algorithm inspired by music	More memory space requirement	Moderate convergence profile
OGHO^25^		Nature based oppositional optimizaion approach	Diversification in population	More complexity
CCO^26^		Nature inspired algorithm	More memory space	Easy implementation
CSA^48^		Based on immune system’s clonal selection theory	Higher efficiency	More computing time
MDE^49^		Mutation or crossover strategies	Easy implementation	Fast convergence
CO^37^		Based on hunting and chasing behaviours of cheetahs	Fast converging capability	Less flexibility
AHA^36^		Intelligent foraging strategies of hummingbirds	Strong robustness	More computational time

### Research gap

Though the previously mentioned approaches have been successfully been applied in various field of power system optimizations, yet these systems have the following disadvantages:

i. Most of the aforesaid techniques have slow convergence rate. Untimely convergence is a major concern for most of the aforementioned methods, resulting in suboptimal solutions and decreased performance and exploration capability.
ii. Many of the aforesaid algorithms stuck in a local optimal region.
iii. Many of the discussed methods have limited ability to explore and exploit.

### Novelty and importance of the work

Novelty and importance of the work are illustrated below:

- Various renewable sources, namely solar, wind and tidal are successfully integrated in the HTS problem to utilize unlimited resources of the said renewable sources.
- Suggested study also integrates electrical vehicle in wind-solar-tidal based HTS systems
- To cope up with these non-linearity, OBL and chaotic phenomena are integrated with MFO in a new approach named COMFO and it is implemented on the proposed work to provide optimal solutions and improve convergence mobility.
- The robustness of the proposed algorithm is accessed by using statistical analysis for both cost and emission minimization problems.

The study utilized two test systems: one comprising 4 hydro units and 3 thermal units, and another featuring 4 hydro units, 3 thermal units, 1 wind unit, 1 solar unit, 1 tidal unit, and 1 EV unit. MFO’s results are compared with existing algorithms, proving its superior performance. This paper’s significant contributions include proposing EVs considering VPPs for tidal-wind-solar-hydro-thermal systems, desirable scheduling based on energy market laws, fast-converging meta-heuristic techniques, and robust MFO algorithms.

### Contributions

The following are the paper’s primary contributions:

- An analysis has been performed to compare the efficacy of the suggested COMFO approach with other efficient optimization methods in order to address its superiority.
- Main contribution of this research work is integrating of solar, wind, tidal and electrical vehicle to strengthen the microgrid as well as provide reliable and pollution free energy supply to mitigate the demand.
- Two single objective functions, such as *i*.*e*. cost reduction and polution reduction have been tested for the four-hydro, three-thermal, one-wind, one-solar, one-tidal, and one-EV unit system.

The rest of the paper is structured as follows: the details of solar, wind, tidal, and electric vehicle (EV) power generation are included in “Modeling of electric vehicle” section. The formulation of a mathematical problem is discussed in “Problem formulation” section. All constraints related to the complex system have been explained in “Constraints” section. The several suggested steps for the proposed optimization technique with a flow chart have been explained in “Algorithm for optimization” section. The results of various simulation-based test system outcomes and statistical evaluation are demonstrated in “Simulation result” section and the research work ended with a conclusion in “Conclusions” section.

**Fig. 1:**
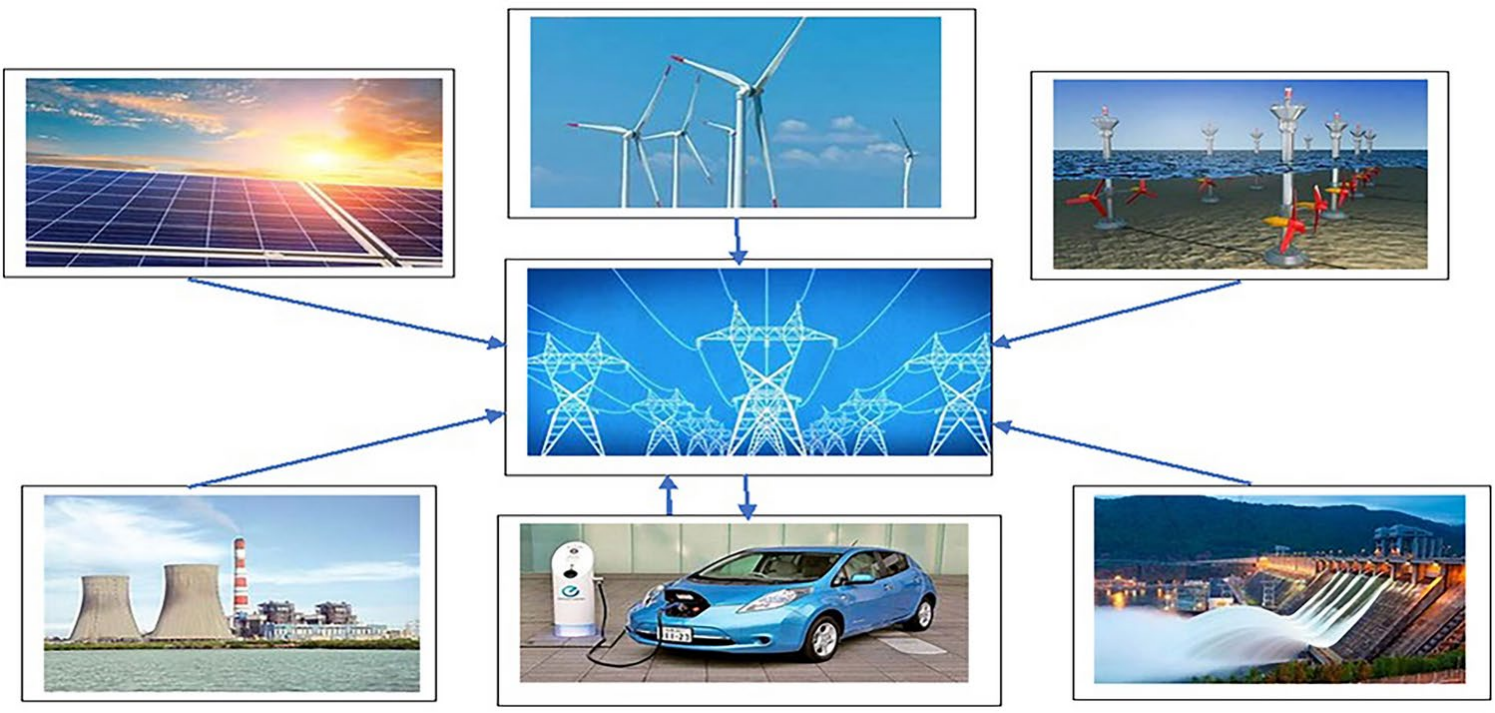
Schematic diagram of solar-wind-hydro-thermal-EV-tidal system.

**Fig. 2:**
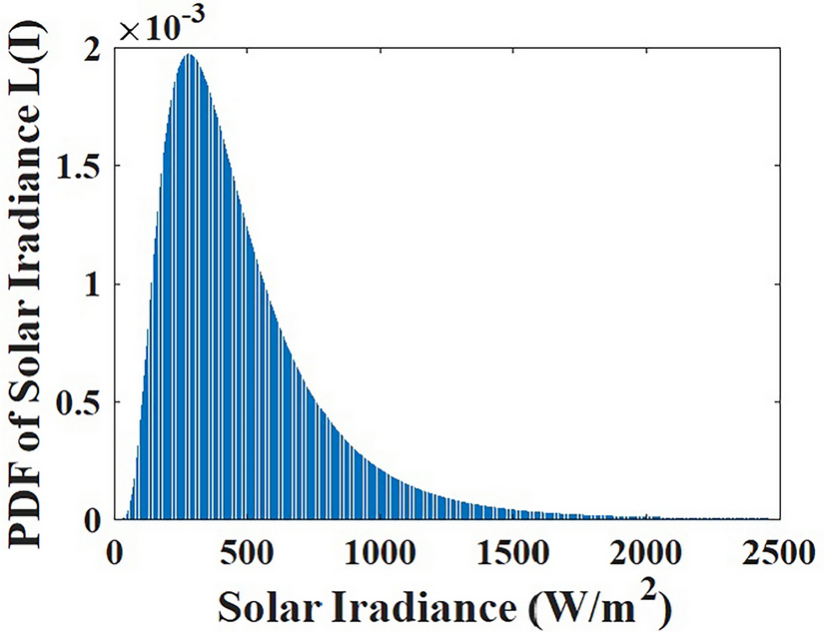
Lognormal based solar irradiance PDF.

**Fig. 3:**
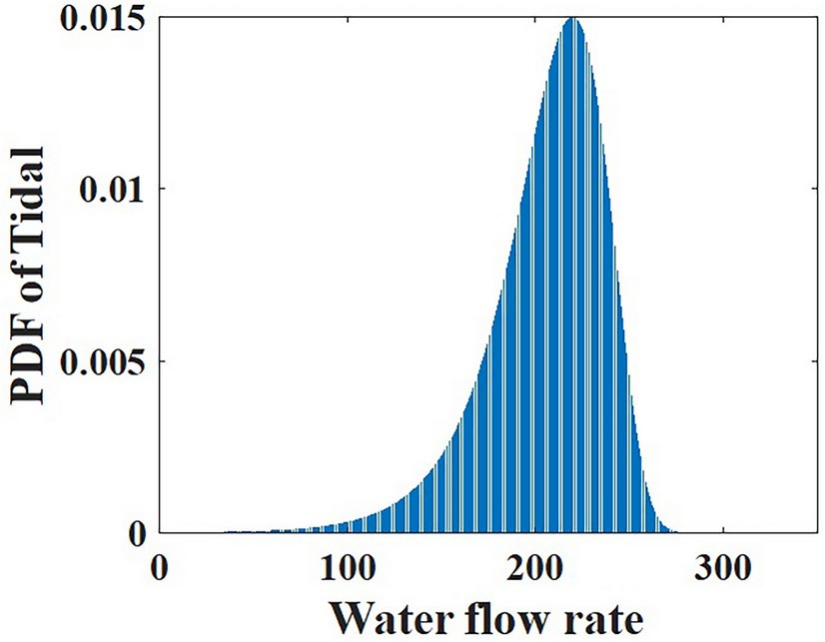
Gumbel based water flow rate PDF.

**Fig. 4:**
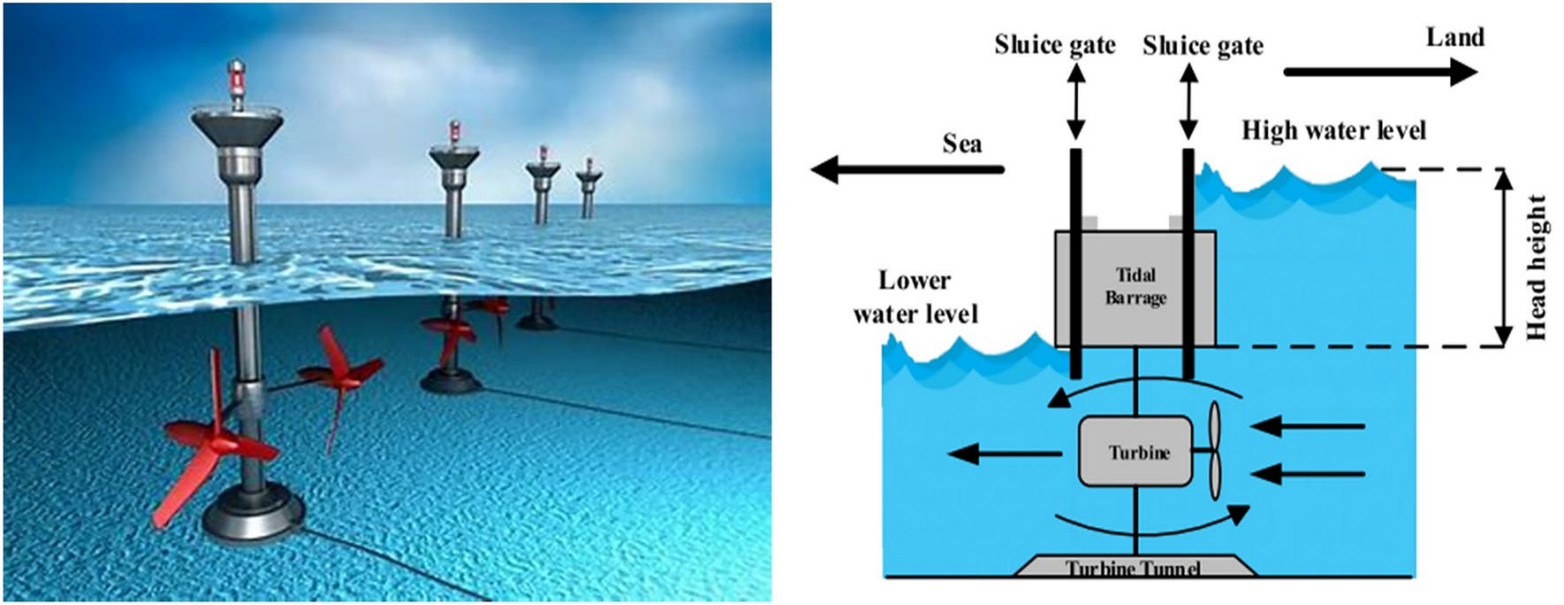
Schamatic diagram of tidal plant.

**Fig. 5:**
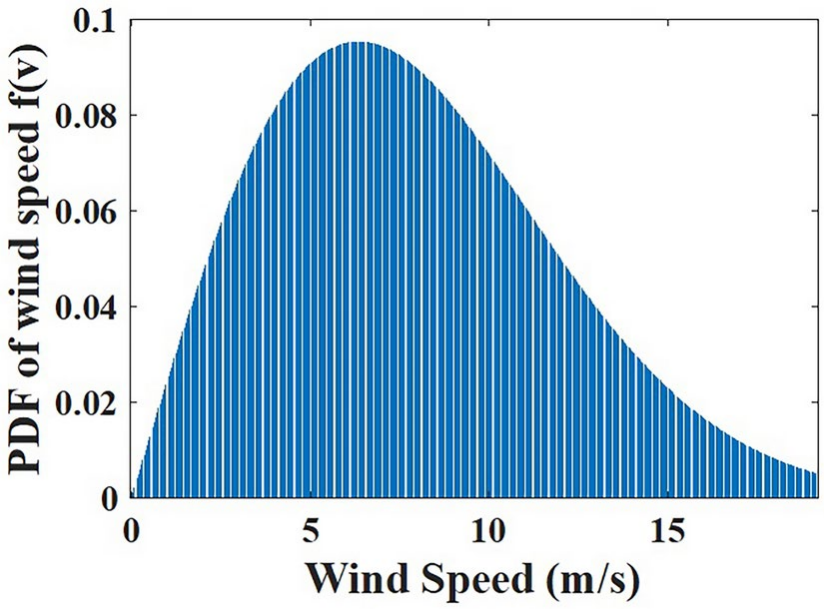
Weibul based wind velocity PDF.

**Fig. 6:**
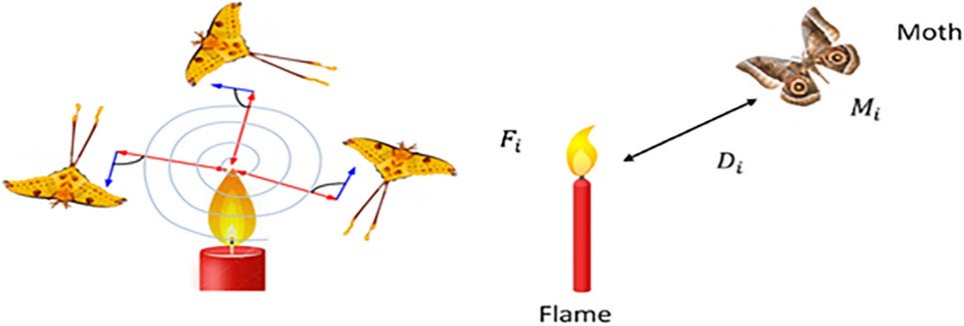
Moth movement as per flame position.

**Fig. 7:**
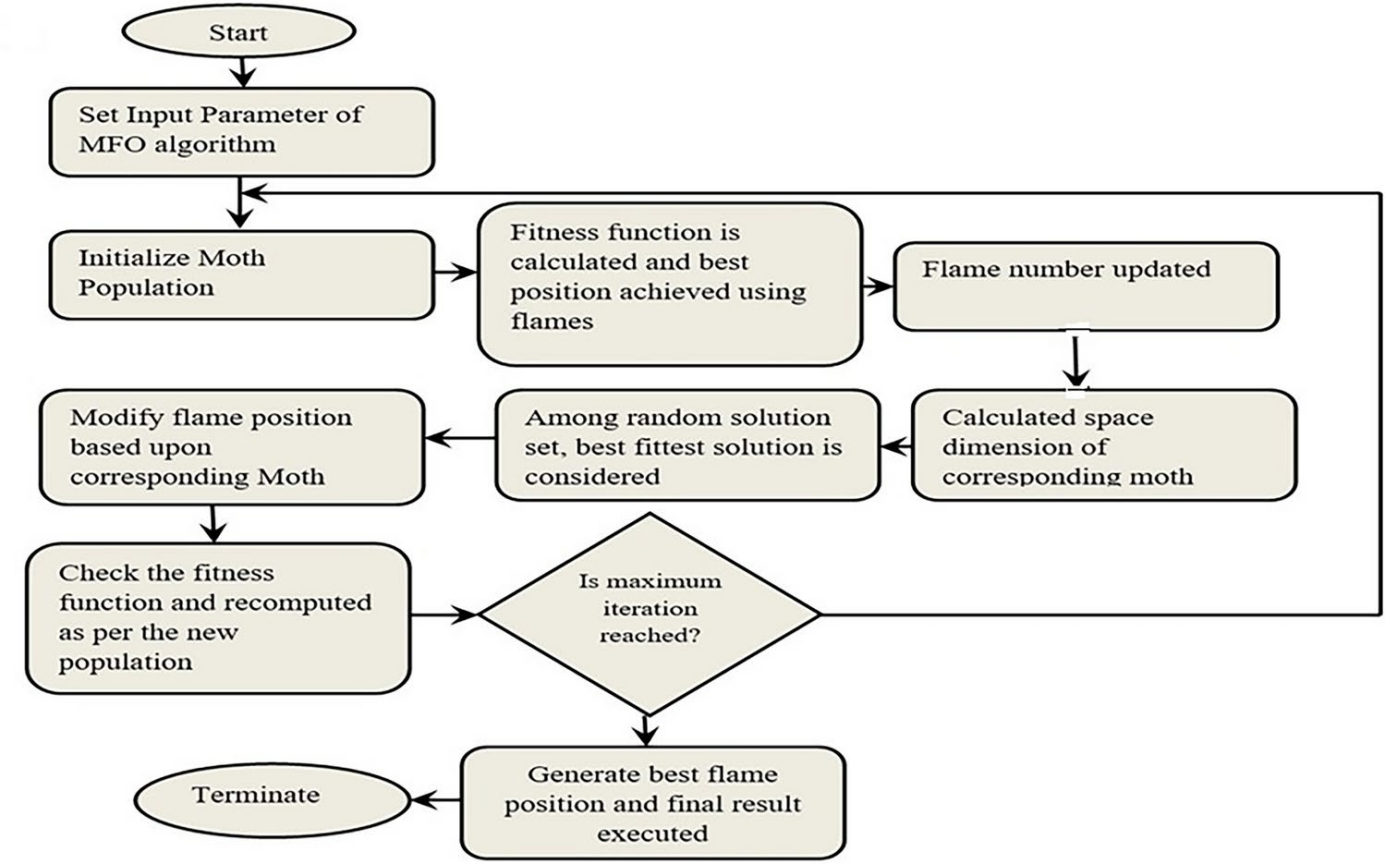
Flow chart of moth flame optimization.

**Fig. 8:**
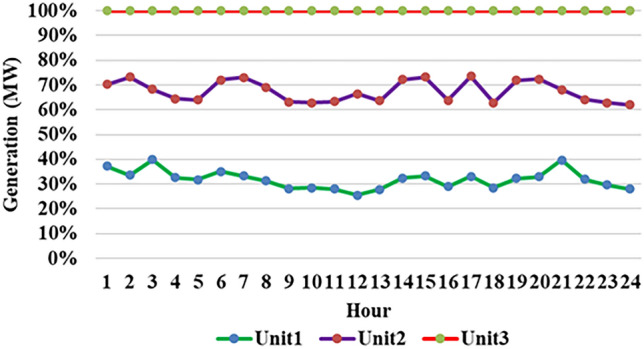
Thermal production of individual plant for 1st test case.

**Fig. 9:**
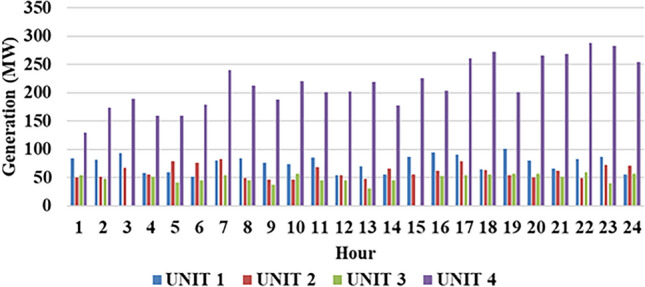
Hydro power production for $2^{nd}$ system at individual hour.

**Fig. 10:**
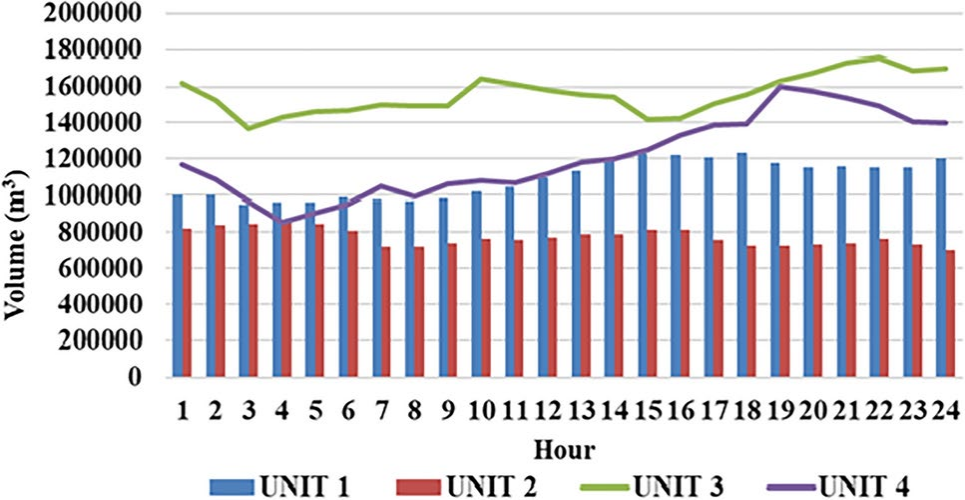
Hourwise hydral volume of water for test system-II.

**Fig. 11:**
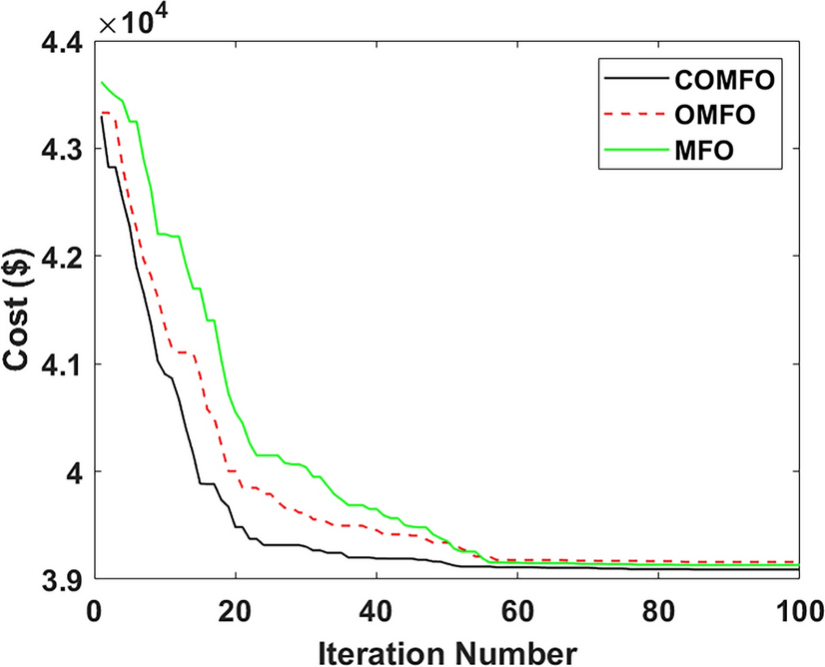
Cost convergence profile for $2^{nd}$ case study.

**Fig. 12:**
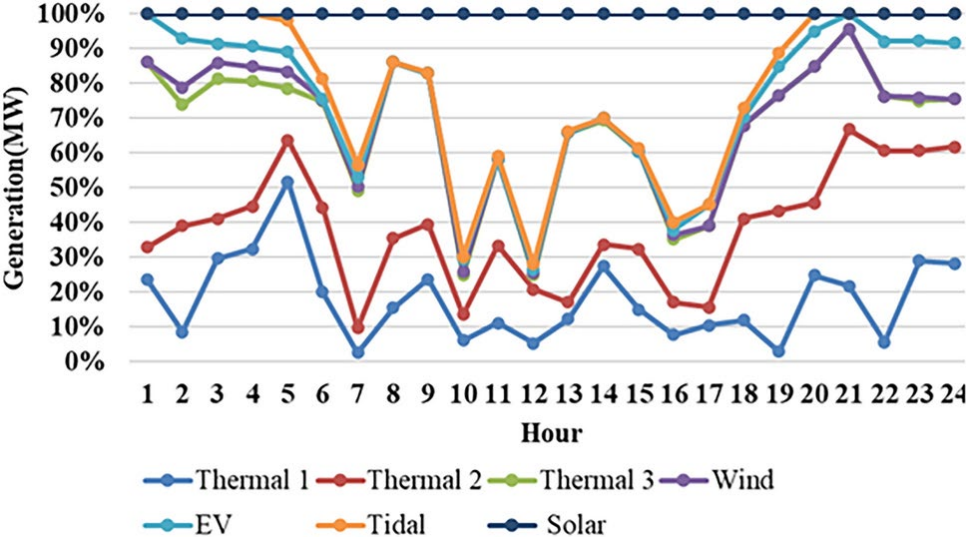
Hour wise thermal, wind, EV, tidal, solar generation.

**Fig. 13:**
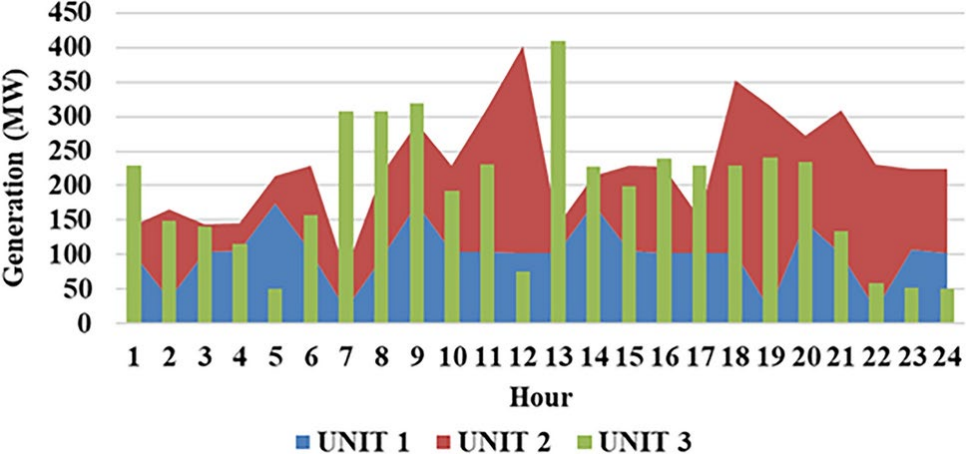
Hour wise power production of $2^{nd}$ case study (Thermal).

**Fig. 14:**
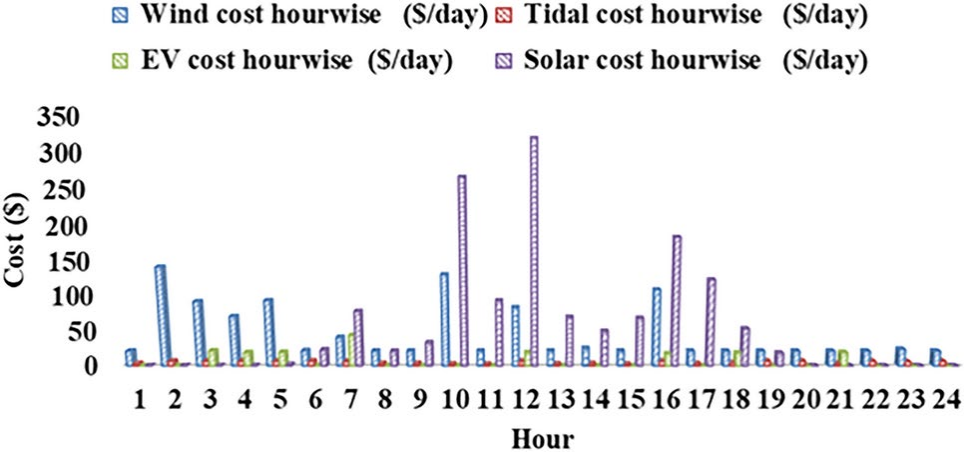
Hour wise cost for wind, solar, tidal, EV.

**Fig. 15:**
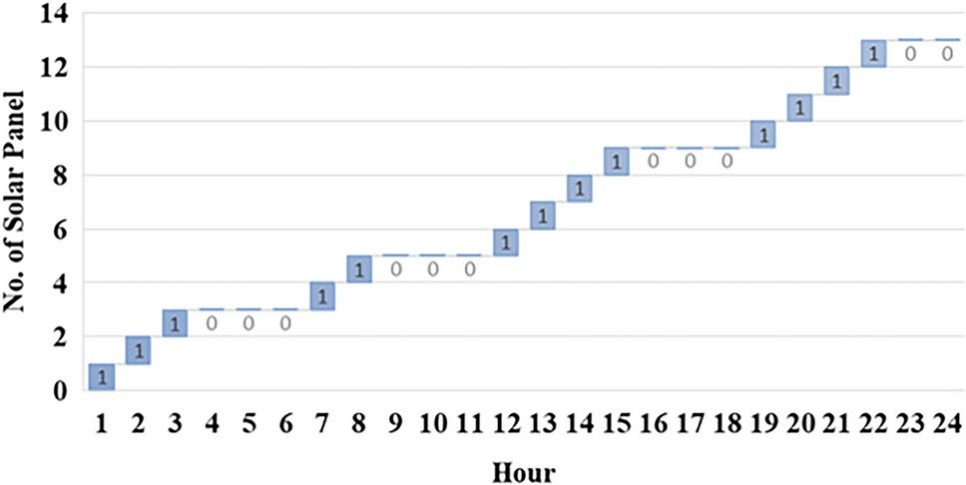
Solar panel on/off status.

## Modeling of electric vehicle

Electric vehicles (EVs) do not rely on fossil fuels to power their batteries, instead using electricity, offering a significant promise for environmental and economic benefits by reducing fossil fuel dependency. EVs play a vital role in vehicle-to-grid (V2G) operations. They can function as both energy sources and loads. Through the Vehicle-to-Grid (V2G) system, electric vehicles (EVs) can connect to the public power grid and supply electricity back to it, aiding in energy demand management. The overall energy consumption of a fleet of EVs is influenced by the individual energy needs of each vehicle, while the fleet’s total energy capacity is determined by the maximum charging capacity of each EV. Adding more EVs to the fleet can increase the available electricity supplied to the grid. Key operational parameters for the EV fleet include the starting and ending points, start and arrival times, as well as the charging and discharging status of each vehicle. Typically, the scheduling of EVs is organized into 24-hour cycles, allowing for an assessment of the time spent on charging, discharging, and driving within this timeframe.

Electric vehicles (EVs) offer significant environmental and economic benefits by reducing reliance on fossil fuels. They use electricity to power their batteries, making them crucial in vehicle-to-grid (V2G) operations. EVs can act as both energy sources and loads, connecting to the public power grid to supply electricity when needed.

Environmental and Economic Benefits: EVs reduce reliance on fossil fuels, lowering emissions and contributing to cleaner air. Economic benefits arise from decreased fuel costs and potential revenue from V2G operations. Through V2G systems, EVs can return electricity to the grid, helping meet energy demands.

Energy Consumption and Capacity: The energy needs of every EV to establish the fleet’s total consumption, while each EV’s maximum charging capacity defines the fleet’s overall energy potential. Additional EVs can provide surplus electricity to the grid.

Operational Parameters: Scheduling time for EVs is typically organized in 24-hour cycles, encompassing charging, discharging, and driving periods. The operational parameters include the starting point, destination, start time, arrival time, and the EV’s charging and discharging status.

24-Hour Cycle Assessment: The duration spent on charging, discharging, and driving an EV is assessed over this 24-hour timeframe. This model allows for efficient energy management and optimal utilization of EVs within the V2G framework, contributing to a more sustainable and reliable power system.

Vehicle-to-Grid (V2G) System: In the V2G system, EVs play a dual role. When connected to the grid, they can absorb excess power during low demand periods and power back to the grid during peak demand times. This two-way energy flow improves grid stability and efficiency. The V2G system also provides an opportunity for EV owners to monetize their vehicles’ unused energy, creating an additional income stream.

Charging and Discharging Dynamics: The patterns of charging and discharging for electric vehicles (EVs) play a vital role in their effective integration into the power grid. Smart charging infrastructure allows for EVs to be charged during off-peak hours when electricity is more affordable and plentiful. In contrast, during periods of high demand, EVs can discharge their stored energy back into the grid, helping to bolster energy supply and lessen reliance on traditional power generation methods.

Impact on the Power Grid: Integrating EVs into the power grid necessitates careful planning and coordination. Grid operators need to account for the combined load from multiple EVs, manage energy distribution effectively, and ensure that the grid can accommodate the fluctuating demands of EV charging and discharging. To facilitate this, advanced software and communication technologies are utilized to monitor and regulate the interactions between EVs and the grid, ensuring smooth operations and preventing overloads.

### Probabilistic model of EV

The capacity of EVs to store energy is a critical aspect of this study. To estimate the available energy, a stochastic model is proposed. The probability density function (PDF) below illustrates that the vehicle-to-grid (V2G) power follows a normal distribution:

1 $$\begin{aligned} {{f}_{{{P}_{car}}}}\left( {{P}_{car}}\right) =\displaystyle \frac{1}{\sqrt{2\pi {{\sigma }^{2}}}}{{e}^{- ^{{{\left( {{P}_{car}}-m\right) }^{2}}}/ _{2{{\sigma }^{2}}}}} \end{aligned}$$

where ${{f}_{{{P}_{car}}}}\left( {{P}_{car}}\right)$ corresponds the PDF of the EV unit’s power output; *m* is mean and ${\sigma }$ isthe normal distribution function’s standard deviation.

### Power and state of charge estimation of EVs

Electric vehicles (EVs) provide the grid with electricity during peak load and consume the grid’s electricity during valley load. The time that EVs spend charging, discharging, and driving can be represented by the length of a 24-hour period. The following two formulas represent EV charging and discharging power.

2 $$\begin{aligned} P_{I,t}^{ch\arg e}= & -\sum \limits _{v=1}^{{{N}_{l}}}{Minimum\left( 0,{{E}_{car,t}} \right) } \end{aligned}$$

3 $$\begin{aligned} P_{I,t}^{disch\arg e}= & \sum \limits _{v=1}^{{{N}_{l}}}{Maximum\left( 0,{{E}_{car,t}} \right) } \end{aligned}$$

Vehicle numbers indicate fleet size and it is represented by ${{N}_{l}}$; Fleet index is represented by *I*; *t* is the time index; ${{E}_{car,t}}$ symbolizes charging and discharging ability.

Depending on the battery’s state of charge (SOC), which is calculated by dividing its capacity by its current state of charge, EV may accelerate a car. Moreover, for reducing losses, SOC guards against excessive charging and battery drain. The SOC is depicted as follows.

4 $$\begin{aligned} \begin{array}{ll} & SO{{C}_{car,t}}=SO{{C}_{init}}-\displaystyle \frac{1}{{{C}_{car}}}\sum \limits _{q=1}^{t}{\left[ {Minimum\,\left( 0,{{E}_{car,q}}\right) }\times {{{\eta }_{ch\arg ing}}} \right] } \\ & \,\,\,\,\,\,\,\,\,\,\,\,\,\,\,\,\, -\displaystyle \frac{1}{{{C}_{car}}}\sum \limits _{q=1}^{t}{\left[ \,Maximum\left( 0,{{E}_{car,q}}\right) \times {{{\eta }_{disch\arg ing}}}+E_{car,q}^{drv} \right] } \end{array} \end{aligned}$$

SOC at $t_{th}$ hour *t* is denoted by ${{soc}_{car,t}}$. In EV, battery ${{\eta }_{ch\arg ing}}$ and ${{\eta }_{disch\arg ing}}$ represent the effectiveness of charging and discharging. Driving force at ${{q}_{th}}$ hour is depicted by $E_{car,q}^{driving}$. The starting value of the charging state is represented by ${{soc}_{initial}}$. ${{C}_{car}}$ represents the EV battery’s capacity.

### Cost calculation of EVs

Cost expenses related to driving a car may be formulated as: and it is represented as follows:

5 $$\begin{aligned} {{Cost}_{EVl}}\left( {{P}_{EVl}}\right) ={{Cost}^{d}_{EVl}}+{{Cost}^{O}_{EVl}}+{{Cost}^{U}_{EVl}} \end{aligned}$$

where ${{Cost}^{O}_{EVl}},~{{Cost}^{d}_{EVl}}$ and ${{Cost}^{U}_{EVl}}$ are the overestimation cost, the direct cost and the underestimation cost.

#### EV direct cost

EV’s direct cost can be calculated in this way:

6 $$\begin{aligned} {{Cost}^{d}_{EVl}}={d^{EV}_l}{{P}_{EVshl}},\,\,\,\,\,where\,\,\,\,\,\,l=1,2,3..,{{n}_{Ev}} \end{aligned}$$

where ${{n}_{EV}}$: number of EV units; ${d^{EV}_l}$: direct cost coefficients;${{P}_{EVshl}}$ is the EV’sscheduled power.

#### EV overestimation cost

Overestimation cost of EV may be defined as follows:

7 $$\begin{aligned} {\left\{ \begin{array}{ll} & {{Cost}^{O}_{EVl}}=\int \limits _{0}^{{{P}_{EVshl}}}{{PF}^{O}_{EVl}}\left( {{P}_{EVl}}-{{P}_{EVshl}}\right) .{{f}_{{{P}_{EV}}}}\left( {{P}_{EVl}}\right) d{{P}_{EVl}} \\ & =\displaystyle \frac{{{PF}^{O}_{EVl}}{\sigma }}{\sqrt{2}\pi }\left( {{e}^{- ^{{{m}^{2}}}/ _{2{{\sigma }^{2}}}}}-{{e}^{- ^{{{\left( m-{{P}_{EVshl}}\right) }^{2}}}/ _{2{{\sigma }^{2}}}}}\right) +\\ & \displaystyle \frac{{{PF}^{O}_{EVl}}}{2}\left( m-{{P}_{EVshl}}\right) \times \left[ Gf\left( \displaystyle \frac{m}{\sqrt{2}\sigma }\right) -Gf\left( \displaystyle \frac{m-{{P}_{EVshl}}}{\sqrt{2}\sigma } \right) \right] \\ \end{array}\right. } \end{aligned}$$

where ${{PF}^{O}_{EVl}}$ is the overestimated component of penalty factor EV.

#### EV underestimation cost

Using V2G power, the underestimation (*i*.*e*. for desired power is less than the available EV power) penalty cost is computed as follows:

8 $$\begin{aligned} {\left\{ \begin{array}{ll} & {{Cost}^{U}_{EVl}}=\int \limits _{{P}_{EVshl}}^{+\infty }{{PF}^{U}_{EVl}}\left( {{P}_{EVl}}-{{P}_{EVshl}}\right) \times {{{f}_{{{P}_{EV}}}}\left( {{P}_{EVl}}\right) d{{P}_{EVl}}} \\ & =\displaystyle \frac{{PF}^{U}_{EVl}}{2}\left( m-{{P}_{EVshl}}\right) \times \left[ 1+Gf\left( \displaystyle \frac{m-{{P}_{EVshl}}}{\sqrt{2}\sigma } \right) +\displaystyle \frac{{{PF}^{U}_{EVl}}.\sigma }{\sqrt{2\pi } }{{e}^{-\displaystyle \frac{{{\left( m-{{P}_{EVshl}}\right) }^{2}}}{2{{\sigma }^{2}}}}}\right] \\ \end{array}\right. } \end{aligned}$$

### Solar photo-voltaic panel designing and cost calculation

The probability distribution function based on lognormal solar irradiance is illustrated in Fig. 2. Equation (9) demonstrates how solar irradiance ${{i}_{rd}}$ generates solar power.

9 $$\begin{aligned} {{f}_{solar}}({{i}_{rd}})=\displaystyle \frac{1}{{{i}_{rd}}d\sqrt{2\pi }}e^{ \displaystyle \frac{-{{\left( \ln {{i}_{rd}}-M \right) }^{2}}}{{{(2d)}^{2}}} }\,\,\,\,for\,\,\,\,\,\,{{i}_{rd}}>0 \end{aligned}$$

Power output is depicted as a function solar irradiance ${{i}_{rd}}$.

10 $$\begin{aligned} \begin{array}{ll} P_{solar}& ={{P}_{sr}}\left( \displaystyle \frac{{{i}_{rd}}^{2}}{{{i}_{rd,sd}}{{R}_{C}}}\right) \,\,\,\,\,\,\,\,\,\,\,\,\,\,for\,\,\,\,\,\,\,\,0<{{i}_{rd}}<{{R}_{C}} \\ & ={{P}_{sr}}\left( \displaystyle \frac{{{i}_{rd}}}{{{i}_{rd,sd}}}\right) \,\,\,\,\,\,\,\,\,\,\,\,\,\,\,\,\,\,for\,\,\,\,\,\,\,\,{{i}_{rd}}>{{R}_{C}} \\ \end{array} \end{aligned}$$

where ${{S}_{R}}$ and *S* are the solar unit’s rating and output power; Two indicators namely standard irradiance and particular irradiance point are ${{i}_{rd,sd}}$ (=1000 $w/m^2$) and ${{R}_{C}}$ (=150 $w/m^2$).

#### Solar cost calculation

Overall solar cost is illustrated as under:^8^:

11 $$\begin{aligned} {{Cost}_{solarl}}\left( {{P}_{solarl}}\right) ={{Cost}^{d}_{solarl}}+{{Cost}^{O}_{solarl}}+{{Cost}^{U}_{solarl}} \end{aligned}$$

Here direct, overestimation and underestimation costs are denoted with ${{Cost}^{d}_{solarl}}$, ${{Cost}^{O}_{solarl}}$ and ${{Cost}^{U}_{solarl}}$ of the ${l^{th}}$ solar unit.

*Solar direct cost* Direct costs of a PV unit may be formulated as under:

12 $$\begin{aligned} {{Cost}^{d}_{solarl}}={d^{solar}_l}{{P}_{solarshl}},\,\,\,\,\,where\,\,\,\,\,\,l=1,2,3..,{{n}_{s}} \end{aligned}$$

Here, ${d^{s}_l}$ represents coefficients of direct costs and ${{P}_{solarshl}}$ schedule power of the ${l^{th}}$ solar.

*Solar underestimation cost* The underestimating cost of the ${l^{th}}$ solar unit may be depicted as:

13 $$\begin{aligned} \begin{array}{ll} {{Cost}^{U}_{solarl}}& ={{PF}^{U}_{solarl}}\left( {{P}_{solaravl}}-{{P}_{solarshl}}\right) \\ & =\,{{PF}^{U}_{solarl}}\int \limits _{{{P}_{solarshl}}}^{{{P}_{solarrl}}}{\left( P_{s}-{{P}_{solarshl}}\right) {{f}_{p_{s}}}(P_{solar})~dP_{Solar}} \\ \end{array} \end{aligned}$$

where ${{P}_{srl}}$ and ${{PF}^{U}_{sl}}$ are the rated power and underestimation panalty cost.

*Solar overestimation cost* If the amount of solar power available is less than what is scheduled, the overestimation cost may be defined as under:

14 $$\begin{aligned} \begin{array}{ll} {{Cost}^{O}_{solarl}}& ={{PF}^{O}_{solarl}}\left( {{P}_{solarshl}}-{{P}_{solaravl}}\right) \\ & ={{PF}^{O}_{solarl}}\int \limits _{0}^{{{P}_{solarshl}}}{\left( {{P}_{solarshl}}-P_{solar}\right) {{f}_{P_{solar}}}(P_{solar})~dP_{solar}} \\ \end{array} \end{aligned}$$

Here ${{f}_{p_{s}}}(P_{solar})$: power output; ${{P}_{solarshl}}$: scheduled power; ${{P}_{solaravl}}$:average power and ${{PF}^{O}_{solarl}}$: Over-estimation panalty cost coefficient.

#### Model of tidal power

The probability model of discharge rate ${{q}_{tidal}}$ in the tidal range is defined by using the Gumbel distribution, as indicated in Eq. (15) and shown in Fig. 3.

15 $$\begin{aligned} {{F}_{qtidal}}\left( {{q}_{tidal}}\right) =\displaystyle \frac{1}{\mu }{{e}^{\left( \displaystyle \frac{{{q}_{tidal}}-\gamma }{\mu }\right) }}{{e}^{\left( -{{e}^{\left( \displaystyle \frac{{{q}_{tidal}}-\gamma }{\mu }\right) }}\right) }} \end{aligned}$$

The display of the tidal power plant can be found in Fig. 4. An equation can be used to mathematically model the tidal range’s output power.

16 $$\begin{aligned} {{P}_{tidal}}({{q}_{tidal}})=\rho g{{q}_{tidal}}h\eta \end{aligned}$$

where ${\rho }$ is the water density ${(\hbox {kg}/\hbox {m}^3)}$, ${q}_{tidal}$ is the discharge value (${({\hbox {m}^3}/\hbox {s})}$) across the turbine, g is the gravity acceleration ${(\hbox {m}/\hbox {s}^2)}$, nh is the distinction between water levels at high and low levels, $\eta$ is the turbine efficiency. These system parameters are configured as h= 3.2 m, $\eta =0.85$, $\rho =1025$ ${\hbox {kg}/{\hbox {m}}^3}$ and g = 9.81 ${(\hbox {m}/\hbox {s}^2)}$. One active power-generating device was found to be the tidal energy system (TES). The modeling methodology in the TES generates the overestimation and underestimation cost models.

17 $$\begin{aligned} & {\left\{ \begin{array}{ll} & \operatorname {Cos}t_{tidal.i}^{O}=C_{tidal.i}^{o}({{P}_{tdls.i}}-{{P}_{tdlav.i}}) \\ & \,\,\,\,\,\,\,\,\,\,\,\,\,\,\,\,\,\,\,\,=C_{tdl.i}^{o}*{{F}_{tdl}}({{P}_{tdlav.i}}<{{P}_{tdls.i}})* \\ & [{{P}_{tdls.i}}-E(({{P}_{tdlav.i}}<{{P}_{tdls.i}})] \\ \end{array}\right. } \end{aligned}$$

18 $$\begin{aligned} & {\left\{ \begin{array}{ll} & \operatorname {Cos}t_{tidal.i}^{U}=C_{tidal.i}^{U}({{P}_{tdlav.i}}-{{P}_{tdls.i}}) \\ & \,\,\,\,\,\,\,\,\,\,\,\,\,\,\,\,\,\,\,\,=C_{tdl.i}^{U}*{{F}_{tdl}}({{P}_{tdlav.i}}>{{P}_{tdls.i}})*\\ & [E({{P}_{tdlav.i}}>{{P}_{tdls.i}})-{{P}_{tdlav.i}}] \\ \end{array}\right. } \end{aligned}$$

where $\operatorname {Cos}t_{tidal.i}^{U}$ and $\operatorname {Cos}t_{tidal.i}^{O}$ represents underestimation cost and overestimation cost of tidal power; scheduled tidal power signifies by ${{P}_{tdls.i}}$. Uncertainty cost coefficient denoted by $C_{tidal.i}^{o}$ and $C_{tidal.i}^{U}$; and available tidal power shown by ${{P}_{tdlav.i}}$ respectively; generated extra and less tidal power denoted with ${{P}_{tdlav.i}}>{{P}_{tdls.i}}$ and ${{P}_{tdlav.i}}<{{P}_{tdls.i}}$.

### Overview of wind energy and battery

#### WP model

The Weibull PDF^46^ is often used to describe the wind speed. It is given by

19 $$\begin{aligned} {{F}_{rand}}({{V}_{wind}})=\displaystyle \frac{k}{d}{{\left( \displaystyle \frac{{{V}_{wind}}}{d} \right) }^{k-1}}\times {{{{e}}^{ -{{\left( \displaystyle \frac{{{V}_{wind}}}{d} \right) }^{k}}}}} \end{aligned}$$

where the chances of getting wind velocity of ${{V}_{wind}}$ is ${{F}_{rand}}$; The shape factor is indicated by $k>0$, while the scale factor is shown by $d>0$. The corresponding cumulative density function (CDF) is represented as:

20 $$\begin{aligned} {{f}_{rand}}({{V}_{wind}})=1-{{{e}}^{ -{{\left( \displaystyle \frac{{{V}_{wind}}}{d} \right) }^{k}} }} \end{aligned}$$

The WP output of the wind unit is given by:

21 $$\begin{aligned} P_{wind}= {\left\{ \begin{array}{ll} & 0\,\,\,\,\,\,\,\,\,\,\,\,\,\,\,\,\,\,\,\,\,\,\,\,\,\,\,\,\,\,\,{{V}_{wind}}<{{V}_{in}}\,or\,\,{{V}_{wind}}>{{V}_{out}} \\ & \displaystyle \frac{{{P}_{wrated}}\left( {{V}_{wind}}-{{V}_{in}}\right) }{{{V}_{rated}}-{{V}_{in}}}\,\,\,\,\,\,\,\,\,{{V}_{in}}\le {{V}_{wind}}<{{V}_{rated}} \\ & {{P}_{wrated}}\,\,\,\,\,\,\,\,\,\,\,\,\,\,\,\,\,\,\,\,\,\,\,\,\,\,\,\,{{V}_{rated}}\le {{V}_{wind}}<{{V}_{out}} \\ \end{array}\right. } \end{aligned}$$

where the indicated output power and the rated power are $P_{wind}$ and ${{P}_{wrated}}$ respectively; ${{V}_{rated}}$ indicates the rated wind velocity; ${{V}_{in}}$ and ${{V}_{out}}$ are used to indicate the cut-in and cut-out velocity of the wind. PDF for WP is as follows:

22 $$\begin{aligned} {{F}_{P_{wind}}}\left( P_{wind}\right) = \displaystyle \frac{ku}{d{{P}_{wrated}}}{{\left( \displaystyle \frac{{{V}_{in}}+u\displaystyle \frac{P_{wind}}{{{P}_{wrated}}}}{d} \right) }^{k-1}}\times {{{e}} ^{ -{{\left( \displaystyle \frac{{{V}_{in}}+u\displaystyle \frac{P_{wind}}{{{P}_{wrated}}}}{d} \right) }^{k}} }} \end{aligned}$$

where $$u={{V}_{rated}}-{{V}_{in}}$$

When $P_{wind}$ = 0 or $P_{wind}$ =${{P}_{wrated}}$, the probability distributions are presented as :

23 $$\begin{aligned} & \begin{array}{ll} {{S}_{rated}}\left( P_{wind}=0\right) & ={{S}_{rated}}\left( V<{{V}_{in}}\right) +{{S}_{rated}}\left( V>{{V}_{out}}\right) \\ & =1-e^{ -{{\left( \displaystyle \frac{{{V}_{in}}}{d} \right) }^{k}}}+e^{ -{{\left( \displaystyle \frac{{{V}_{out}}}{d} \right) }^{k}}}\\ \end{array} \end{aligned}$$

24 $$\begin{aligned} & \begin{array}{ll} {{S}_{rated}}(P_{wind}={{P}_{wrated}})\,\,& ={{S}_{rated}}\left( {{V}_{rated}}\le V<{{V}_{out}}\right) \\ & = e^{ -{{\left( \displaystyle \frac{{{V}_{rated}}}{d} \right) }^{k}} }-e^{-{{\left( \displaystyle \frac{{{V}_{out}}}{d} \right) }^{k}}} \\ \end{array} \end{aligned}$$

The corresponding $P_{wind}$ CDF is expressed as:

25 $$\begin{aligned} {{f}_{P_{wind}}}(P_{wind})= {\left\{ \begin{array}{ll} & 0\,\,\,\,\,\,\,\,\,\,\,\,\,\,\,\,\,\,\,\,\,\,\,\,\,\,\,\,\,\,\,\,\,\,\,\,\,\,\,\,\,\,\,\,\,\,\,\,\,\,\,\,\,\,\,\,\,\,\,\,\,\,\,\,\,\,\,\,\,\,\,\,\,\,\,\,\,\,\,\,\,\,\,\,\,\,\,\,\,\,\,\,\,\,\,\,\,\,\,\,\,\,\,\,\,\,\,\,\,\,\,\,\,\,\,\,\,\,\,\,\,\,\,P{wind}<0 \\ & \displaystyle \frac{ku}{d{{P}_{wrated}}}{{\left( \displaystyle \frac{{{V}_{in}}+u\displaystyle \frac{P_{wind}}{{{P}_{wrated}}}}{d} \right) }^{k-1}}\times {e^{ -{{\left( \displaystyle \frac{{{V}_{in}}+u\displaystyle \frac{P_{wind}}{{{P}_{wrated}}}}{d} \right) }^{k}} }}\,\,\,\,\,\,\,\,\,\,\,\,\,\,\,\,\,\,0\le P_{wind}<{{P}_{wrated}} \\ & 1\,\,\,\,\,\,\,\,\,\,\,\,\,\,\,\,\,\,\,\,\,\,\,\,\,\,\,\,\,\,\,\,\,\,\,\,\,\,\,\,\,\,\,\,\,\,\,\,\,\,\,\,\,\,\,\,\,\,\,\,\,\,\,\,\,\,\,\,\,\,\,\,\,\,\,\,\,\,\,\,\,\,\,\,\,\,\,\,\,\,\,\,\,\,\,\,\,\,\,\,\,\,\,\,\,\,\,\,\,,\,\,\,\,\,\,\,\,\,P_{wind}\ge {{P}_{wrated}} \\ \end{array}\right. } \end{aligned}$$

#### WP cost computation

It is necessary to integrate wind power into the current electricity network during periods of high demand. When WP must be implemented with the current power system, two cost categories are taken into account: overestimation and underestimation. This is because wind electricity generation has an intrinsic unpredictable nature. To forecast wind power generation in this context, the Weibull distribution is employed. Weibull-based PDF is shown in Fig. 5. The overall cost for wind electricity generation is expressed as

26 $$\begin{aligned} \begin{array}{ll} & {{TotalCost}_{wind}}={\sum \limits _{m=1}^{{{N}_{wind}}}{{Cost}_{windm}}\left( {{P}_{windm}}\right) } \\ & = {\sum \limits _{m=1}^{{{N}_{wind}}}{\left( {{Cost}^{d}_{windm}}+{{Cost}^{O}_{windm}}+{{Cost}^{U}_{windm}} \right) }} \\ \end{array} \end{aligned}$$

where ${{TotalCost}_{wind}}$: total wind cost; ${{N}_{wind}}$: number of wind units; ${{Cost}^{d}_{windm}}$: direct cost; ${{Cost}^{O}_{windm}}$: overestimation cost; ${{Cost}^{U}_{windm}}$: underestimation cost; *m* represents unit indices.

*Direct cost* For ${{m}^{th}}$ WP unit, direct cost is given by:

27 $$\begin{aligned} {{Cost}^{d}_{windm}}={d^{wind}_m}{{P}_{windm}},\,\,\,\,\,where\,\,\,\,\,\,m=1,2,3..,{{n}_{w}} \end{aligned}$$

Here, ${d^{wind}_m}$ is direct cost coefficients and ${{P}_{windm}}$ is power scheduled from ${m^{th}}$ unit.

*Overestimation cost* There is discussion of the cost of overestimation when power generation is less than expected. This suggests that there will be insufficient WP to satisfy the required demand. When the load need is met, the additional power will come from the spinning reserve. The cost of the overestimation can be calculated using (28).

28 $$\begin{aligned} {\left\{ \begin{array}{ll} & {{Cost}^{O}_{windm}}={{Pf}^{O}_{windm}}\times {{P}_{windm}}\left[ 1-e^{ -{{\left( \displaystyle \frac{{{V}_{in}}}{{{s}}} \right) }^{{{j}}}}} +e^{ -{{\left( \displaystyle \frac{{{V}_{out}}}{{{s}}} \right) }^{{{j}}}}}\right] + \\ & \left( \displaystyle \frac{{{P}_{wratedm}}{{V}_{in}}}{{{V}_{rated}}-{{V}_{in}}}+{{P}_{windm}} \right) \left[ e^{ -{{\left( \displaystyle \frac{{{V}_{in}}}{{{c}}} \right) }^{{{j}}}}}-e^{ -{{\left( \displaystyle \frac{{{V}_{in}}+{{P}_{windm}}\displaystyle \frac{{{V}_{rated}}-{{V}_{in}}}{{{P}_{wrated}}}}{{{s}}} \right) }^{{{j}}}} } \right] \\ & \,+\left( \displaystyle \frac{{{P}_{wrated}}{s}}{{{V}_{rated}}-{{V}_{in}}}\right) \left[ \zeta \left\{ 1+\displaystyle \frac{1}{{{j}}},{{\left( \displaystyle \frac{{{V}_{in}}+{{P}_{windm}}\displaystyle \frac{{{V}_{rated}}-{{V}_{in}}}{{{P}_{wrated}}}}{{{s}}} \right) }^{{{j}}}} \right\} -\zeta \left\{ 1+\displaystyle \frac{1}{{{j}}},{{\left( \displaystyle \frac{{{V}_{in}}}{{{s}}} \right) }^{{{j}}}} \right\} \right] \\ \end{array}\right. } \end{aligned}$$

*Underestimation cost* The costs of underestimation occur when the actual WP exceeds projections. Wind turbines will store any excess electrical energy they generate in batteries, as they would otherwise lose their power. As shown in (29), is the formula used to determine the underestimate cost:

29 $$\begin{aligned} {\left\{ \begin{array}{ll} & {{Cost}^{U}_{windm}}= {{Pf}^{U}_{windm}}\times \left( {{P}_{wrated}}-{{P}_{windm}} \right) \left[ e^{ -{{\left( \displaystyle \frac{{{V}_{rated}}}{{{s}}} \right) }^{{{j}}}}}-e^{ -{{\left( \displaystyle \frac{{{V}_{out}}}{{{s}}} \right) }^{{{j}}}}} \right] + \\ & \left( \displaystyle \frac{{{P}_{wrated}}{{V}_{in}}}{{{V}_{rated}}-{{V}_{in}}}+{{P}_{windm}} \right) \left[ e^{ -{{\left( \displaystyle \frac{{{v}_{rated}}}{{{s}}} \right) }^{{{j}}}} }-e^{ -{{\left( \displaystyle \frac{{{V}_{in}}+{{P}_{windm}}\displaystyle \frac{{{v}_{rated}}-{{v}_{in}}}{{{P}_{wrated}}}}{{{s}}} \right) }^{{{j}}}}} \right] \\ & +\displaystyle \frac{{{P}_{wrated}}{{s}}}{{{V}_{rated}}-{{V}_{in}}}\left[ \zeta \left\{ 1+\displaystyle \frac{1}{{{j}}},{{\left( \displaystyle \frac{{{V}_{in}}+{{P}_{windm}}\displaystyle \frac{{{V}_{rated}}-{{V}_{in}}}{{{P}_{wrated}}}}{{{s}}} \right) }^{{{j}}}} \right\} -\zeta \left\{ 1+\displaystyle \frac{1}{{{j}}},{{\left( \displaystyle \frac{{{V}_{rated}}}{{{s}}} \right) }^{{{j}}}} \right\} \right] \\ \end{array}\right. } \end{aligned}$$

Here, ${{Cost}^{O}_{windm}}$ and ${{Cost}^{U}_{windm}}$: ${m^{th}}$ wind unit’s overestimation and underestimation costs respectively; ${{P}_{wrated}}$ and ${{V}_{rated}}$: rated output power and rated velocity respectively; ${{V}_{in}}$ and ${{V}_{out}}$: cut-in and cut-out velocity of wind turbine respectively; ${{Pf}^{U}_{windm}}$ and ${{Pf}^{O}_{windm}}$: underestimation and overestimation cost co-efficient.

## Problem formulation

Incorporating wind, solar, tidal, and electric vehicles (EVs) into the optimal operation of hydrothermal power systems introduces a highly complex and non-linear optimization challenge. To accurately model this system under real-world conditions, several technical constraints and factors must be taken into account. These include the unique operational characteristics of renewable sources such as wind, solar, and tidal as well as those of conventional thermal and hydro units. Additional complexities such as valve point effects, prohibited operating zones (POZ) and transmission losses are also integrated into the problem formulation. These constraints are essential for evaluating the performance and efficiency of the hybrid test system (HTS), which combines these diverse energy sources. The following sections outline the objective functions and critical constraints-both equality and inequality-that are vital to optimizing the wind-solar-tidal-EVs-based HTS model. These considerations are key to ensuring that the system operates efficiently while adhering to the technical and practical limitations of each energy source.

### Objective function

The primary goal of this challenge is to reduce the cost of power generation and create a world free of pollutants.

#### Case 1: HTS without wind, solar and EVs

In the HTS problem, wind, solar and electric vehicles are not taken into account, and the objective function of thermal units has a quadratic cost function. In addition, the cost of producing hydroelectric power plants is very low. Only water discharges from hydro reservoirs have been used. Thus, for the thermal generating unit, the objective function of the HTS problem is provided by:

30 $$\begin{aligned} Min\ C=\sum \limits _{i=1}^{{{N}_{p}}}{{{C}_{pi}}({{P}_{pi}})} \end{aligned}$$

The cost function of thermal power is explained by the quadratic equation, which is shown in Eq. (31):

31 $$\begin{aligned} {{C}_{pi}}({{P}_{pi}})\ ={{\alpha }_{pi}}{{({{P}_{pi}})}^{2}}+{{\beta }_{pi}}{{P}_{pi}}+{{\gamma }_{pi}} \end{aligned}$$

where the cost function’s coefficients of the ${{i}^{th}}$ thermal power are represented by ${{\alpha }_{pi}}$, ${{\beta }_{pi}}$ and ${{\gamma }_{pi}}$. In Eq. (32), the impact of the valve point is taken into account. It illustrates a sinusoidal feature.

32 $$\begin{aligned} \begin{array}{ll} {{C}_{pi}}({{P}_{pi}})=& {{\alpha }_{pi}}{{({{P}_{pi}})}^{2}}+{{\beta }_{pi}}{{P}_{pi}}+{{\gamma }_{pi}}\\ & +\left| {{\delta }_{pi}}\ Sin\ ({{\varepsilon }_{pi}}\times (P_{pi}^{\min }-{{P}_{pi}}) \right| \end{array} \end{aligned}$$

#### Case 2: HTS with wind, solar, EVs, tidal and energy storage

An equation (33) describes the cost function of the HTS problem based on solar, wind and electric vehicles.

33 $$\begin{aligned} \begin{array}{ll} Min\ C= & \sum \limits _{i=1}^{{{N}_{p}}}{{{C}_{pi}}\left( {{P}_{pi}}\right) }+\sum \limits _{a=1}^{{{N}_{w}}}{{{x}_{wa}}}\left( {{P}_{wa}}\right) +\sum \limits _{l=1}^{{{N}_{s}}}{{{C}_{srl}}}\left( {{P}_{srl}}\right) \\ & +\sum \limits _{m=1}^{{{N}_{v}}}{{{C}_{vehm}}}\left( {{P}_{vehm}}\right) +\sum \limits _{i=1}^{{{N}_{tidal}}}{{{Cost}_{tidali}}}\left( {{P}_{tidali}}\right) \end{array} \end{aligned}$$

In the above equation, ${{X}_{wa}}\left( {{P}_{wa}}\right)$ represents the wind generation cost; The cost of solar and electric vehicle generation are represented by ${{C}_{slr}}\left( {{P}_{slr}}\right)$ and ${{C}_{vehm}}\left( {{P}_{vehm}}\right)$, respectively; The number of wind unit, solar panel, and electric vehicle fleets is represented by ${{N}_{w}}$, ${{N}_{s}}$ and ${{N}_{v}}$, respectively.

## Constraints

### Power balance constraints

#### HTS without REs and EV

The hydro-thermal scheduling problem’s power balance constraint^7^ in the absence of renewable energy sources and EVS is provided by:

34 $$\begin{aligned} \sum \limits _{i=1}^{{{N}_{p}}}{{{C}_{pi}}\left( {{P}_{pi}}\right) }+\sum \limits _{i=1}^{{{N}_{hd}}}{{{P}_{hd,i}}}={{P}_{de}}+{{P}_{loss}} \end{aligned}$$

${{N}_{hd}}$ is the total number of hydroelectric plants; ${{P}_{de}}$ is the network’s total power demand; ${{P}_{loss}}$ is the sum of transmission losses; ${{P}_{hd,i}}$ is the production of hydropower and is mostly determined by the storage volume and discharge rate, which can be explained as follows:

35 $$\begin{aligned} {{P}_{hd,i}}={{\lambda }_{1i}}{{\left( {{V}_{i}} \right) }^{2}}+{{\lambda }_{2i}}{{\left( {{Q}_{i}} \right) }^{2}}+{{\lambda }_{3i}}{{V}_{i}}{{Q}_{i}}+{{\lambda }_{4i}}{{V}_{i}}+{{\lambda }_{5i}}{{Q}_{i}}+{{\lambda }_{6i}} \end{aligned}$$

where, $\lambda$: hydro power plant generation coefficients. ${V}_{i}$, ${{Q}_{i}}$: reservoir storage volumes for hydro power plant.

#### HTS with REs and EV

When using EVs and renewable energy sources, the hydro-thermal scheduling problem’s power balance constraint is provided by:

36 $$\begin{aligned} \begin{array}{cc} & \sum \limits _{i=1}^{{{N}_{p}}}{{{C}_{pi}}\left( {{P}_{pi}}\right) }+\sum \limits _{i=j}^{{{N}_{hd}}}{{{P}_{hd,j}}}+\sum \limits _{a=1}^{{{N}_{w}}}{{{x}_{wa}}}\left( {{P}_{wa}}\right) +\sum \limits _{l=1}^{{{N}_{s}}}{{{C}_{srl}}}\left( {{P}_{srl}}\right) \\ & +\sum \limits _{m=1}^{{{N}_{v}}}{{{C}_{vehm}}}\left( {{P}_{vehm}}\right) +\sum \limits _{i=1}^{{{N}_{tidal}}}{{{Cost}_{tidali}}}\left( {{P}_{tidali}}\right) ={{P}_{de}}+{{P}_{loss}} \end{array} \end{aligned}$$

### Inequality constraints

#### Generation limit constraints

The lower and upper range of thermal power, hydro power, wind power, solar power and EV are shown by Eqs. (37)–(42):

37 $$\begin{aligned} P_{pi}^{\min }\le & {{P}_{pi}}\le P_{pi}^{\max } {where, i=1,2,3,...,{{N}_{p}}} \end{aligned}$$

38 $$\begin{aligned} P_{hd}^{\min }\le & {{P}_{hd}}\le P_{hd}^{\max } {where, j=1,2,3,...,{{N}_{hd}}} \end{aligned}$$

39 $$\begin{aligned} P_{wa}^{\min }\le & {{P}_{wa}}\le P_{wa}^{\max } where, a=1,2,3,...,{{N}_{w}} \end{aligned}$$

40 $$\begin{aligned} P_{srl}^{\min }\le & {{P}_{srl}}\le P_{srl}^{\max } where, l=1,2,3,...,{{N}_{s}} \end{aligned}$$

41 $$\begin{aligned} P_{veh}^{\min }\le & {{P}_{veh}}\le P_{veh}^{\max } where, m=1,2,3,...,{{N}_{v}} \end{aligned}$$

42 $$\begin{aligned} P_{tidali}^{\min }\le & {{P}_{tidali}}\le P_{tidali}^{\max } where, i=1,2,3,...,{{N}_{tidal}} \end{aligned}$$

Lower and upper power limits of ${{i}^{th}}$ thermal power unit is presented by ${P_{pi}^{min }}$, ${P_{pi}^{max }}$; $P_{hd}^{min }$, $P_{wa}^{min }$, $P_{srl}^{min }$ and $P_{veh}^{min }$ are the lower power generation of ${{j}^{th}}$ hydro, ${{a}^{th}}$ wind, ${{l}^{th}}$ solar and ${{m}^{th}}$ EV units;$P_{hd}^{max }$, $P_{wa}^{max }$, $P_{slr}^{max }$ and $P_{veh}^{max }$ are presented upper generation of ${{j}^{th}}$ hydro, ${{a}^{th}}$ wind, ${{l}^{th}}$ solar and ${{m}^{th}}$ EV units.

The schematic diagram of hydro-thermal-wind-PEV- tidal-solar system is presented in Fig. 1.

#### Power limits for EV charging and discharging

43 $$\begin{aligned} {{\text {x}}_{\text {j,k}}}= {\left\{ \begin{array}{ll} -{{E}_{ch\arg ing(\max )}}\le {{E}_{v,t}}<0, & charging \\ {{E}_{disch\arg ing\left( \max \right) \,}}\ge {{E}_{v,t}}>0, & discharging \\ {{E}_{v,t}}\,=\,0, & driving \\ \end{array}\right. } \end{aligned}$$

#### Limit of state of charging for EVs

44 $$\begin{aligned} SO{{C}_{\min }}\le SO{{C}_{v,t}}\le SO{{C}_{\max }} \end{aligned}$$

#### Initial and final SOC limit

45 $$\begin{aligned} SO{{C}_{0}}=SO{{C}_{T}}=SO{{C}_{initial}} \end{aligned}$$

### Restrictions on hydro power

#### Limits on reservoir storage volumes

46 $$\begin{aligned} {{V}_{i,\min }}\le {{V}_{i}}\le {{V}_{i,\max }} \end{aligned}$$

where, ${{V}_{i,\min }},{{V}_{i,\min }}$ are the boundaries of the storage volume of the ${{i}^{th}}$ reservoir.

#### Limits on water release

47 $$\begin{aligned} {{Q}_{i,\min }}\le {{Q}_{i}}\le {{Q}_{i,\max }} \end{aligned}$$

where, ${{Q}_{i,\min }},\,\,{{Q}_{i,\max }}$ are the water release’s lowest and maximum limits of the ${{i}^{th}}$ power plant.

#### Reservoir restrictions for initial and final reserve volumes

Each unit’s reservoir storage opening and closing shall adhere to this restriction.

48 $$\begin{aligned} V_{i}^{0}=V_{i}^{bn} \end{aligned}$$

49 $$\begin{aligned} V_{i}^{t}=V_{i}^{end} \end{aligned}$$

where, $V_{i}^{0}$ and $V_{i}^{t}$ are the ${{i}^{th}}$ hydro unit’s tank storage at time period 0 and *t*; $V_{i}^{bn}$ and $V_{i}^{end}$ are the beginning and ending the hydro unit’s tank storage constraints *i*.

#### Water restrictions and dynamic balance

The hydro plant’s reservoir storage must continuously monitor the hydraulic system’s continuity equations when it is compressed by spills and inflows at the previous event and it is expressed as follows^48^:

50 $$\begin{aligned} V_{i}^{t}=V_{i}^{t-1}+I_{i}^{t}-Q_{i}^{t}-S_{i}^{t}+\sum \limits _{n=1}^{{{N}_{us}}}{\left( {{Q}_{n,t-{{\tau }_{n,us}}}}+{{S}_{n,t-{{\tau }_{n,us}}}} \right) } \end{aligned}$$

The ${{i}^{th}}$ hydro units inflow and spillage are depicted using $I_{i}^{t},\,\,S_{i}^{t}$; *n*, *us* is the upstream component; ${{\tau }_{n,us}}$ is the time delay.

51 $$\begin{aligned} \begin{array}{ll} Minimum\,{{emission}_{pou}}\\ =\sum \limits _{t=1}^{T}{\sum \limits _{i=1}^{{{N}_{pou}}}{\left[ {{b}_{i0}}+{{b}_{i1}}P_{poui}^{t}+{{b}_{i2}}{{(P_{poui}^{t})}^{2}}+{{b}_{i3}}\exp ({{b}_{i4}}P_{poui}^{t}) \right] }}\end{array} \end{aligned}$$

In (51), ${{b}_{i0}}$, ${{b}_{i1}}$, ${{b}_{i2}}$, ${{b}_{i3}}$ and ${{b}_{i4}}$ denote emission coefficients whereas $P_{poui}^{t}$ represents thermal generation.

## Algorithm for optimization

Moth flame optimization (MFO) is a well-known algorithm based on swarm intelligence, inspired by the navigation behavior of moths. The optimization technique mimics the moth’s ability to navigate using a method known as transverse orientation, which is especially noticeable during nighttime. This natural behavior allows moths to maintain a constant angle with respect to the moonlight as they fly, helping them reach their destination. By applying this concept, the searching capabilities of moths are enhanced in the optimization process. MFO, originally introduced by Mirjalili, utilizes this navigation mechanism to solve complex optimization problems. The moths’ movements are simulated to find optimal solutions by constantly adjusting their position in search of a better solution. The working steps of the MFO algorithm are often illustrated using a flowchart, which details the step-by-step process of this meta-heuristic algorithm. Mirjali^50^ is the one who initially invented this meta-heuristic algorithm. The placements of the moths have mostly been moved to the vicinity of the best solutions, and the flame sequence has been altered based on the best solutions. The moth population set’s location is shown by:

52 $$\begin{aligned} {{M}_{o}}^{@}=\left[ \begin{array}{llll} m{{o}^{@}}_{1,1} & m{{o}^{@}}_{1,2} & ...\begin{array}{ll}. & .....m{{o}^{@}}_{1,d} \\ \end{array} \\ ... & ... & \begin{array}{ll}... & ... \\ \end{array} \\ \begin{array}{ll}... \\ m{{o}^{@}}_{k,1} \\ \end{array} & \begin{array}{ll}... \\ m{{o}^{@}}_{k,2} \\ \end{array} & \begin{array}{ll} \begin{array}{ll}... \\ ....... \\ \end{array} & \begin{array}{ll}... \\ ....m{{o}^{@}}_{k,d} \\ \end{array} \\ \end{array} \\ \end{array} \right] \end{aligned}$$

Here, *d* indicates the dimension of the variables and *k* denotes the moths number (*i*.*e*. size of population).The following array is used to store the appropriate fitness.

53 $$\begin{aligned} C{{m}^{@}}=\left[ \begin{array}{ll} C{{m}_{1}}^{@} \\ \begin{array}{ll} C{{m}_{2}}^{@} \\ \begin{array}{ll}. \\ . \\ \end{array} \\ \end{array} \\ C{{m}_{n}}^{@} \\ \end{array} \right] \end{aligned}$$

where $C{{m}_{i}}$ is the function of fitness of the ${{i}^{th}}$ moth.

Accordingly, the flames can also be displayed in a matrix form, as presented below:

54 $$\begin{aligned} {{F}_{l}}^{@}=\left[ \begin{array}{lll} f{{l}^{@}}_{1,1} & f{{l}^{@}}_{1,2} & ...\begin{array}{ll}. & .....f{{l}^{@}}_{1,D} \\ \end{array} \\ ... & ... & \begin{array}{ll}... & ... \\ \end{array} \\ \begin{array}{ll}... \\ f{{l}^{@}}_{n,1} \\ \end{array} & \begin{array}{ll}... \\ f{{l}^{@}}_{n,2} \\ \end{array} & \begin{array}{ll} \begin{array}{ll}... \\ ....... \\ \end{array} & \begin{array}{ll}... \\ ....f{{l}^{@}}_{n,D} \\ \end{array} \\ \end{array} \\ \end{array} \right] \end{aligned}$$

The flame equivalent array is introduced to accumulate the corresponding fitness values as illustrated below:

55 $$\begin{aligned} O{{F}^{@}}=\left[ \begin{array}{ll} \begin{array}{ll} OF_{1}^{@} \\ OF_{2}^{@} \\ \end{array} \\ . \\ \begin{array}{ll}. \\ OF_{n}^{@} \\ \end{array} \\ \end{array} \right] \end{aligned}$$

where, ${{i}^{th}}$ flame fitness value is shown by $OF_{1}^{@}$.

Three-tuple estimation is added by the MFO algorithm’s ordinary surface. Thus, the primary approach is included as follows:

56 $$\begin{aligned} MF=[T_{R1}^{@},T_{R2}^{@},T_{R3}^{@}] \end{aligned}$$

An important factor in changing the moths’ position is a logarithmic spiral. Moths are shifted in the direction of the flame by using navigation or transverse orientation steps. Lastly, moths are moved forward and backward in the vicinity of flames in a logarithmic spiral motion. The following formula can be used to depict the logarithmic spiral movement:

57 $$\begin{aligned} S{{I}^{@}}(M_{i}^{@},F_{j}^{@})={{l}_{i}}\times {{e}^{nq}}\times \cos (2\pi q)+F_{j}^{@} \end{aligned}$$

where *n* signifies a constant number depending on where a moth is closest to the appropriate flame, it has a logarithmic spiral structure. ${{j}^{th}}$ flame of the ${{i}^{th}}$ moth length is presented using ${{l}_{i}}$; the range of *q* is (−1 to 1) and it is a random value; The outcome of ${{l}_{i}}$ moth can be represented as follows:

58 $$\begin{aligned} {{l}_{i}}=\left| {{F}_{l}}_{j}^{@}-MO_{i}^{@} \right| \end{aligned}$$

To increase the action qualities surrounding the flames of succeeding moths, the iteration process is further performed.

59 $$\begin{aligned} fla\begin{array}{lll} & cou & \\ \end{array}=\begin{array}{ll} & \\ \end{array}round\left[ HM-{{r}_{q}}\times \displaystyle \frac{HM-1}{{{Y}_{\max }}} \right] \end{aligned}$$

where, the largest quantity of flames is shown using *HM*; the maximum number of iterations is determined by ${{Y}_{\max }}$ and Iterations in progress can be recognized by ${{r}_{q}}$. Movement of moth according to the flame is shown in Fig. 6. Flow chart of chaotic oppositional moth flame optimization is shown in Fig. 7.

### Chaotic based learning (CBL)

Many evolutionary algorithms are inspired by random initialization and the ongoing search for the optimal solution. MFO still cannot find the global optimal solution better than other approaches, which also affects the rate of convergence. To reduce this impact, MFO and chaos behavior are combined to generate COMFO. Faster overall searches are made possible by chaos’s unpredictable and non-repeating characteristics, which be crucial for accelerating a metaheuristic algorithm’s convergence.

The CMFO approach integrates various chaotic maps with MFO to regulate its parameters. Ten chaotic maps with various behaviors have been considered. In the range of 0 to 1, the starting value for the optimal solution is 0.7. Table 2 discusses the different chaotic maps.

**Table 2:** List of various chaotic maps.

Sl. No.	Name	Chaotic map
K1	Sine	${{X}_{i+1}}=a/4\left( sin\prod x \right)$
K2	Gussian map	$~{{r}_{k+1}}={{r}_{k+1}}\left\{ 0\ ,\ {{r}_{k}}=0\ ,\ \displaystyle \frac{1}{{{r}_{k}}}\bmod \ (1)=\displaystyle \frac{1}{{{r}_{k}}}-\left[ \displaystyle \frac{1}{{{r}_{k}}} \right] \right.$
K3	Circle	${{r}_{k+1}}={{r}_{k+b}}-(a/2\pi )sin\left( 2\pi k\right) mod\left( 2 \right)$
K4	Cubic	${{r}_{j+1}}=a{{r}_{j}}\left( 1-{{r}_{j}}^{2} \right)$
K5	Chebyshev map	$~{{r}_{j+1}}=\cos \left( k{{\cos }^{-1}}\left( {{r}_{k}}\right) \right)$
K6	Sinusoidal	${{X}_{i+1}}=a\left( {{X}_{i}} \right) 2\left( sin\prod {{x}_{i}} \right)$
K7	Tent	$$\begin{array}{ll} X_{i+1}& = {\left\{ \begin{array}{ll} \displaystyle \frac{{{X}_{i}}}{0.7}; {{\text {X}}_{i}}<0.7 \\ \displaystyle \frac{10}{3}\text {(}1-{{X}_{i}}\text {) }; {{\text {X}}_{i}}\ge 0.7 \\ \end{array}\right. } \end{array}$$
K8	Liebovitch map	$~{{r}_{k+1}}=a{{r}_{k}}\left( 1-{{r}_{k}} \right)$
K9	Iterative map	${{\text {r}}_{\text {k}+\text {1}}}=\text {Sin}\left( \displaystyle \frac{\text {a }\!\!\pi \!\!}{\text {rk}} \right) \text {, }\!\!\alpha \!\!\in \left( \text {U,1} \right)$
K10	Logistic map	$~{{r}_{k+1}}=a{{r}_{k}}\left( 1-{{r}_{k}} \right)$

### Opposite number

The mirror position of the proposed solution uses the opposite number (60). The equivalent opposite number ${{X}_{o}}$ of a randomly generated candidate solution with interval [a, b] for a one-dimensional search space is represented as:

60 $$\begin{aligned} {{X}_{o}}=a+b-X \end{aligned}$$

where the search space’s minimum and maximum limits are *a* and *b*, respectively. The preceding statement is stated similarly for n-dimensional search space by the following Eq. (61)

where the lowest and maximum bounds of the search space are *a* and *b*, respectively. The following Eq. (61) similarly states the above statement for a n-dimensional search space:

61 $$\begin{aligned} {{X}_{ok}}={{a}_{k}}+{{b}_{k}}-{{X}_{k}} \end{aligned}$$

where $$k=1,2,....,n$$ and $${{X}_{k}}={{X}_{1}},{{X}_{2}},....,{{X}_{n}}$$

### Jumping rate

Jumping rate offers a new approach that exceeds the current one in terms of fitness value (62). Fresh solutions are developed using the jumping rate equation, and the quasi-opposite solution has been determined. The algorithm receives assistance in determining the optimal solution globally.

62 $$\begin{aligned} {{j}_{R}}=({{j}_{R,Max}}-{{j}_{R,Min}})-({{j}_{R,Max}}-{{j}_{R,Min}})\left( \displaystyle \frac{{{f}_{Max}}-f}{{{f}_{Max}}} \right) \end{aligned}$$

where ${{j}_{R}}$ is jumping rate; ${{j}_{R,Max}}$ denotes maximum jumping rate; minimum jumping rate is denoted by ${{j}_{R,Min}};$ *f* is function for current iteration and ${{f}_{Max}}$ is maximum number of iteration.

### Steps of COMFO for wind-solar-EV-tidal-energy storage based HTS problem

Step 1: Initialization
  The hybrid power system’s input characteristics are taken into consideration, including the fuel cost coefficients, emission coefficients, water discharge rate of hydro plants, solar PV, hydro, wind, and thermal generators, as well as the input parameters of electric vehicles.
  The outputs are randomly selected within the specified search space.
  Inequality constraints are checked to confirm the viability of the generated results. If any non-elite solutions are found, they are reinitialized.
  A possible solution array is constructed based on the population size of the moths, which is given in matrix form.
Step 2: Non-dominated sorting
  A non-dominated sorting Pareto front is incorporated depending on the moths’ population matrix.
  Each solution is compared with others to determine if it is dominated. If no other feasible solution dominates a given solution, it is considered a non-dominated Pareto optimal front.
  The validation process ensures that at least one objective function value is better for the non-dominated solution.
    63 $$\begin{aligned} {{f}_{i}}(m)\le {{f}_{i}}({{m}^{'}}) ,\,\,\,\ i=1,2....,P \end{aligned}$$
    The equation below holds true for at least one value of *i*.
    64 $$\begin{aligned} {{f}_{i}}(m)<{{f}_{i}}({{m}^{'}}) \end{aligned}$$
    where ${{f}_{i}}(m)$ is the $i^{th}$ objective function and $m=({{M}_{1}},....,{{M}_{n}})$ are the objective functions’ control variables.
Step 3: Fitness representation
  A column array is introduced to represent fitness equivalent values based on each moth’s position, optimizing for cost.
Step 4: Position alternation
  Moths’ positions are modified based on their proximity to the flames, moving them up to the current iteration within the search space.
  The best existing positions of each moth are aligned with the flame positions, ensuring optimal values are achieved.
Step 5: Flame fitness array
  Fitness values are stored in the flame fitness array. Moths’ positions can be adjusted to enhance search space exploration.
  By validating moths and including optimal flame positions towards the end of the search process, the effectiveness of the flame is determined.
Step 6: MFO technique
  Triple estimate functions are used by the MFO approach to determine the optimal solution.
  The lower and higher bounds of the control variable are indicated in the penetration space, where the moths’ fitness values are computed.
Step 7: Compromise solution
  The compromise solution is reached using a pseudo-weight vector, which calculates each solution after optimization.
  The method optimizes for the minimum and maximum values of the target function by allocating weights to each created Pareto solution set.
    65 $$\begin{aligned} M{{O}^{*}}(i,j)=rand(j)*({{U}_{*}}(i)-{{L}_{*}}(i))+{{L}_{*}}(i) \end{aligned}$$
    where, ‘$T_{R2}^{*}$’ denotes the moths movement in the section of the penetrating space. *L* and *U* are the lower and higher limit of the control variable. Procedure for modernization of the recognized matrix *M* is processed in this way.
    66 $$\begin{aligned} T_{R2}^{*}:M{{O}^{*}}\rightarrow M{{O}^{*}} \end{aligned}$$
    If the principle of termination is in accordance then $T_{R3}^{*}$ functions proceeds “yes,” and if the termination standard is not in in accordance it returns “No”. Each flame’s position progression is determined using the equation below:
    67 $$\begin{aligned} MO_{i}^{*}=S{{I}^{*}}\left( MO_{i}^{*},F_{lj}^{*} \right) \end{aligned}$$
    Here, $SI^*$ is the spiral characteristics; $MO_{i}^{*}$ presents the ${{i}^{th}}$ moth of the $F_{lj}^{*}$ flame position.
    The best result is obtained by introducing a logarithmic spiral to update fitness values from the worst to the global best using (54)–(55). Results are boosted based on the iteration count and the flames amount is reduced gradually using equation (68).
Step 8: The pseudo-weight vector is used to obtain the compromise solution, and each solution is produced using:
  68 $$\begin{aligned} \left( w{{v}_{i}} \right) =\displaystyle \frac{[{{f}_{i}}{{(x)}^{\max }}-{{f}_{i}}\left( x \right) /{{f}_{i}}{{(x)}^{\max }}-{{f}_{i}}{{\left( x \right) }^{\min }}]}{\sum \limits _{k=1}^{b}{\left[ {{f}_{k}}{{(x)}^{\max }}-{{f}_{k}}\left( x \right) /{{f}_{k}}{{(x)}^{\max }}-{{f}_{k}}{{\left( x \right) }^{\min }} \right] }} \end{aligned}$$
  The minimum and highest value of objective functions is expressed by ${{f}_{i}}{{(x)}^{\max }}$ and ${{f}_{i}}{{(x)}^{\min }}$.
Step 9: Stopping criteria
  The algorithm terminates and the results are presented if the halting criteria-the maximum number of iterations is met.
    This algorithm will halt its execution if the stopping criteria, or maximum number of iterations, is met and print the result.

### Advantages of COMFO over standard MFO algorithm

Advantages of COMFO over standard MFO algorithm are listed below:

- COMFO effectively handles complex and nonlinear optimization problems.
- Robustness is better than other existing algorithms including MFO.
- It has better exploration and exploitation capability as compared to the conventional MFO approach.
- Multi-objective optimization problems can be dealt in an effective manner.
- It has better Convergence superiority.

## Simulation result

### CEC benchmark system

CEC Benchmark functions consisting of a number of unimodal, multi-modal, hybrid, or composite functions are mostly used to judge the effectiveness of any optimization technique. The aforesaid Benchmark functions are mostly used in various dimensions such as with 10D, 30D, 50D, and 100D dimensions. In the proposed research work, the present authors successfully used IEEE CEC 2017 benchmark functions having 30D and 50D dimensions. The present authors used ${{10}^{4}}\times D$ number of iterations to optimize the aforementioned functions. The present authors evaluate the performance of the algorithm in 30 different runs. There are various groups into which the benchmark functions may be classified. In this study, most widely used benchmark functions namely unimodal (F1–F3), multimodal (F4–F16), hybrid (F17–F22) and composite functions (F23–F30) are used for accessing the efficacy of the COMFO approach.

#### CEC 2017 (30D)

The best mean error values and standard deviations (SD) obtained by the proposed COMFO and other approaches for CEC 2017 with 30D are illustrated in Table 3. Mean error values less than $10 \times 10^{-8}$ are considered zero for all participating algorithms. Table 3 clearly shows that, in terms of mean error values, proposed COMFO outperforms most of the other discussed algorithms used in this work for the majority of test functions. In contrast to the other algorithms, COMFO outperforms in reaching optimal values for unimodal and multimodal test functions. Furthermore, it is observed from the standard deviation values listed in Table 3 that among all the algorithms, the proposed COMFO has the highest level of precision. Table 4 compares the best mean error values and SD generated by different MCTs for hybrid and composite functions. Moreover, the computational results listed in Table 4 demonstrates that the proposed COMFO performs better in terms of mean error values and SD compared to the other approaches. This facts clearly prove that COMFO has the potential to provide most accurate and effective results. The Wilcoxon signed rank test with a significance threshold of 0.05 is used to compare the mean error values of the proposed MCT with the other MCTs for each test function in order to assess statistical significance. Competing MCTs are assigned the “+”, “=”, and “−” signs based on their statistical performance versus the suggested COMFO, as determined by the results of the signed-rank test. If the performance of any algorithm is better, equal to, or worse than the recommended COMFO, it is indicated by the “+”, “=”, and “−” symbols. Table 9 proves the statistical robustness of the proposed COMFO over its rivals, which shows that among the participating MCTs, the proposed MCT receives the most “+” signs. Moreover, the Friedman rank test^51^ is used to assess the proposed MCT’s overall statistical performance. Based on the Friedman rank, the proposed COMFO comes in first place among all approaches.

#### CEC 2017 (50D)

Moreover, to judge the efficacy of the proposed approach for high dimensional problem, CEC 2017 with 50 dimension is used.The best mean error values and standard deviations (SD) for the 50D scenario are shown in Table 4, which has been compiled by the suggested COMFO and other participating MCTs. The competitiveness of the suggested COMFO’s performance across most uni-modal and multi-modal functions is illustrated by the best mean error values displayed in Table 4. Additionally, the SD values show that the proposed strategy performs consistently better than the other strategies considered. According to Table 9, the recommended approach outperforms alternative approaches in terms of mean error values and SD for the majority of hybrid and composite functions. Since the suggested COMFO achieves more “+” signs than the other eligible algorithms, the results of the Wilcoxon signed-rank test listed in Table 5, prove its statistical superiority. Moreover, the Friedman rank test listed in the bottom row of Table 5 clearly demonstrates that the recommended COMFO ranks first among all the discussed algorithms.

**Table 3:** Statistical comparison of COMFO on CEC 2017 with 30D for F1–F16.

CEC 2017 (D=30)								
Function		BWM_HS	CVnew	SGSADE	HGSO	SCA	MFO	COMFO
Unimodal								
F1	Mean	3.798$\times 10^{3}$	1.199$\times 10^{10}$	3.498$\times 10^{-8}$	5.497$\times 10^{3}$	0.000	3.073$\times 10^{-8}$	2.986 × 10^−8^
	SD	4.799$\times 10^{3}$	0.000	3.945$\times 10^{-8}$	1.123$\times 10^{3}$	0.000	2.049$\times 10^{-8}$	2.029 × 10^−8^
	Sign	+	+	$-$	$-$	$-$	$-$	
F3	Mean	1.199$\times 10^{-7}$	1.515$\times 10^{2}$	1.338$\times 10^{2}$	5.958$\times 10^{2}$	2.119$\times 10^{-8}$	3.299$\times 10^{-8}$	1.983 × 10^−7^
	SD	4.499$\times 10^{-8}$	9.459$\times 10^{1}$	1.173$\times 10^{2}$	2.875$\times 10^{2}$	2.198$\times 10^{-8}$	1.920$\times 10^{-8}$	2.137 × 10^−7^
	Sign	+	+	+	+	+	+	
Multi-modal								
F4	Mean	6.799$\times 10^{1}$	1.558$\times 10^{1}$	1.399$\times 10^{1}$	4.729$\times 10^{2}$	4.342$\times 10^{1}$	3.242$\times 10^{-8}$	2.788 × 10^−8^
	SD	3.101$\times 10^{1}$	2.797$\times 10^{1}$	2.598$\times 10^{1}$	3.012$\times 10^{2}$	2.897	3.004$\times 10^{-8}$	1.316 × 10^−8^
	Sign	+	+	+	+	+	$-$	
F5	Mean	5.101$\times 10^{1}$	1.298$\times 10^{2}$	8.901$\times 10^{1}$	6.196$\times 10^{2}$	1.448$\times 10^{1}$	3.711	3.087 × 10^1^
	SD	1.901$\times 10^{1}$	2.801$\times 10^{1}$	1.799$\times 10^{1}$	9.896	2.399	2.693	1.087 × 10^1^
	Sign	+	+	+	+	$-$	$-$	
F6	Mean	1.199$\times 10^{-5}$	2.124$\times 10^{1}$	2.304$\times 10^{-8}$	5.983$\times 10^{2}$	1.101$\times 10^{-8}$	1.115$\times 10^{-8}$	8.230 × 10^−8^
	SD	2.224$\times 10^{-5}$	8.231	1.499$\times 10^{-8}$	7.701	1.502$\times 10^{-8}$	1.318$\times 10^{-8}$	1.234 × 10^−7^
	Sign	$-$	+	$-$	+	$-$	$-$	
F7	Mean	5.988$\times 10^{1}$	2.299$\times 10^{2}$	1.297$\times 10^{2}$	8.398$\times 10^{2}$	4.891$\times 10^{1}$	3.582$\times 10^{1}$	5.417
	SD	9.701	2.099$\times 10^{1}$	1.599$\times 10^{1}$	6.196$\times 10^{1}$	2.252	8.229$\times 10^{-1}$	5.268 × 10^−1^
	Sign	+	+	+	+	+	+	
F8	Mean	4.988$\times 10^{1}$	1.197$\times 10^{2}$	8.289$\times 10^{1}$	8.302$\times 10^{2}$	1.295$\times 10^{1}$	3.416	3.157
	SD	1.303$\times 10^{1}$	2.701$\times 10^{1}$	1.603$\times 10^{1}$	2.604$\times 10^{1}$	2.789	1.777	1.596
	Sign	+	+	+	+	+	=	
F9	Mean	1.099$\times 10^{1}$	2.198$\times 10^{3}$	5.972$\times 10^{-8}$	1.801$\times 10^{3}$	0.199	0.403	0.000
	SD	8.0044$\times 10^{1}$	8.505$\times 10^{2}$	6.033$\times 10^{-8}$	2.402$\times 10^{2}$	0.303	0.842	0.005 × 10^−8^
	Sign	+	+	+	+	+	+	
F10	Mean	2.755$\times 10^{3}$	4.498$\times 10^{3}$	5.099$\times 10^{3}$	5.194$\times 10^{3}$	1.101$\times 10^{3}$	2.463$\times 10^{3}$	4.420 × 10^2^
	SD	4.801$\times 10^{2}$	3.035$\times 10^{2}$	5.499$\times 10^{2}$	3.098$\times 10^{2}$	2.396$\times 10^{2}$	3.543$\times 10^{2}$	9.417 × 10^1^
	Sign	+	+	+	+	+	+	
F11	Mean	9.501$\times 10^{1}$	3.704$\times 10^{1}$	5.036$\times 10^{1}$	1.502$\times 10^{3}$	1.803$\times 10^{1}$	4.206	3.100
	SD	3.199$\times 10^{1}$	1.888$\times 10^{1}$	3.099$\times 10^{1}$	2.901$\times 10^{1}$	2.001$\times 10^{1}$	3.846	1.541
	Sign	+	+	+	+	+	=	
F12	Mean	5.011$\times 10^{5}$	5.099$\times 10^{9}$	1.906$\times 10^{4}$	5.002$\times 10^{4}$	4.199$\times 10^{2}$	4.872$\times 10^{2}$	5.125
	SD	4.501$\times 10^{5}$	5.899$\times 10^{9}$	6.988$\times 10^{3}$	3.098$\times 10^{4}$	1.503$\times 10^{2}$	2.64$\times 10^{2}$	3.632
	Sign	+	+	+	+	+	+	
F13	Mean	1.901$\times 10^{4}$	7.988$\times 10^{1}$	2.987$\times 10^{2}$	5.501$\times 10^{4}$	2.112$\times 10^{1}$	0.901$\times 10^{1}$	7.299 × 10^−1^
	SD	2.197$\times 10^{4}$	2.902$\times 10^{1}$	3.001$\times 10^{2}$	2.099$\times 10^{3}$	0.983$\times 10^{1}$	4.826	4.056 × 10^−1^
	Sign	+	+	+	+	+	+	
F14	Mean	4.011$\times 10^{3}$	5.023$\times 10^{1}$	6.222$\times 10^{1}$	2.299$\times 10^{3}$	1.889$\times 10^{1}$	2.793$\times 10^{1}$	3.146 × 10^−1^
	SD	3.301$\times 10^{3}$	7.099	8.912	1.803	2.501	1.989	0.688 × 10^−1^
	Sign	+	+	+	+	+	+	
F15	Mean	8.112$\times 10^{3}$	3.778$\times 10^{1}$	4.888$\times 10^{1}$	3.812$\times 10^{3}$	4.018	4.561	3.146 × 10^1^
	SD	8.908$\times 10^{3}$	8.803	3.001$\times 10^{1}$	5.012$\times 10^{2}$	2.101	2.897	1.401 × 10^1^
	Sign	+	=	+	+	$-$	$-$	
F16	Mean	4.888$\times 10^{2}$	7.509$\times 10^{2}$	5.054$\times 10^{2}$	3.299$\times 10^{3}$	2.706$\times 10^{1}$	4.222$\times 10^{1}$	5.478
	SD	1.972$\times 10^{2}$	2.112$\times 10^{2}$	1.801$\times 10^{2}$	3.399$\times 10^{2}$	2.978$\times 10^{1}$	5.745$\times 10^{1}$	2.911
	Sign	+	+	+	+	+	+

**Table 4:** Statistical comparison of COMFO, on CEC 2017 with 50D for F17–F30.

CEC-2017 (D=50)								
Function		BWM_HS	CVnew	SGSADE	HGSO	LSHADE-cnEpSin	LSHADE-SPACMA	COMFO
Hybrid								
F17	Mean	3.099$\times 10^{2}$	2.011$\times 10^{2}$	8.099$\times 10^{1}$	2.014$\times 10^{3 }$	3.199$\times 10^{1}$	3.987$\times 10^{1}$	1.810 × 10^1^
	SD	1.889$\times 10^{2}$	6.901$\times 10^{1}$	2.198$\times 10^{1}$	1.983$\times 10^{1}$	4.986	7.401	1.077 × 10^1^
	Sign	+	+	$-$	+	$-$	$-$	
F18	Mean	1.501$\times 10^{5}$	4.009$\times 10^{1}$	1.988$\times 10^{3}$	1.001$\times 10^{4}$	1.986$\times 10^{1}$	3.801$\times 10^{1}$	0.732 × 10^1^
	SD	5.901$\times 10^{4}$	6.985	1.801$\times 10^{3}$	5.712$\times 10^{4}$	6.901$\times 10^{-1}$	2.021	1.666 × 10^−1^
	Sign	+	$-$	=	+	$-$	$-$	
F19	Mean	7.907$\times 10^{3}$	1.897$\times 10^{1}$	2.199$\times 10^{1}$	1.966$\times 10^{3}$	4.512	8.201	7.523 × 10^−1^
	SD	9.902$\times 10^{3}$	3.101	6.190	2.901$\times 10^{3}$	1.901	2.303	6.006
	Sign	+	+	+	+	+	+	
F20	Mean	1.799$\times 10^{2}$	1.812$\times 10^{2}$	0.909$\times 10^{2}$	1.701$\times 10^{3}$	2.512$\times 10^{1}$	7.805$\times 10^{1}$	3.475 × 10^2^
	SD	8.901$\times 10^{1}$	9.615$\times 10^{1}$	4.905$\times 10^{1}$	2.988$\times 10^{2}$	6.501	4.201$\times 10^{1}$	2.042 × 10^1^
	Sign	+	+	+	+	=	+	
F21	Mean	2.604$\times 10^{2}$	1.801$\times 10^{2}$	2.803$\times 10^{2}$	2.899$\times 10^{3}$	1.899$\times 10^{2}$	1.799$\times 10^{2}$	7.236
	SD	1.501$\times 10^{1}$	2.712$\times 10^{1}$	2.199$\times 10^{1}$	2.499$\times 10^{1}$	2.815	3.533	1.813
	Sign	+	+	+	+	+	+	
F22	Mean	1.912$\times 10^{3}$	1.198$\times 10^{3}$	1.801$\times 10^{2}$	3.899$\times 10^{3}$	2.901$\times 10^{2}$	2.612$\times 10^{2}$	1.263 × 10^1^
	SD	1.599$\times 10^{3}$	1.907$\times 10^{3}$	1.199$\times 10^{1}$	8.278$\times 10^{2}$	1.499$\times 10^{1}$	2.901$\times 10^{1}$	7.645
	Sign	+	+	=	+	=	=	
Composite								
F23	Mean	4.111$\times 10^{2}$	3.808$\times 10^{2}$	3.966$\times 10^{2}$	1.977$\times 10^{3}$	2.701$\times 10^{2}$	2.212$\times 10^{2}$	4.047 × 10^1^
	SD	4.889$\times 10^{1}$	4.714	2.692$\times 10^{1}$	5.394$\times 10^{1}$	2.981$\times 10^{1}$	3.502$\times 10^{1}$	2.124
	Sign	+	+	+	+	+	+	
F24	Mean	5.001$\times 10^{2}$	4.502$\times 10^{2}$	3.099$\times 10^{4}$	2.099$\times 10^{3}$	4.098$\times 10^{2}$	1.901$\times 10^{1}$	1.822 × 10^1^
	SD	2.194$\times 10^{1}$	2.601$\times 10^{2}$	2.199$\times 10^{1}$	8.701$\times 10^{1}$	2.515	1.712	2.515 × 10^−1^
	Sign	+	+	+	+	+	+	
F25	Mean	3.901$\times 10^{2}$	3.612$\times 10^{2}$	4.099$\times 10^{2}$	2.866$\times 10^{2}$	2.404$\times 10^{2}$	1.888$\times 10^{1}$	2.036 × 10^1^
	SD	2.401	7.312$\times 10^{-1}$	4.901	2.887$\times 10^{1}$	7.401$\times 10^{-3}$	1.828$\times 10^{-2}$	1.410 × 10^−3^
	Sign	+	+	+	+	+	+	
F26	Mean	2.701$\times 10^{3}$	3.711$\times 10^{2}$	2.912$\times 10^{3}$	4.701$\times 10^{3}$	9.310$\times 10^{2}$	9.831$\times 10^{2}$	1.222 × 10^2^
	SD	6.401$\times 10^{2}$	3.201$\times 10^{1}$	2.101$\times 10^{2}$	1.889$\times 10^{2}$	4.701$\times 10^{1}$	3.498$\times 10^{1}$	2.757 × 10^1^
	Sign	+	+	+	+	+	+	
F27	Mean	5.618$\times 10^{2}$	5.301$\times 10^{2}$	5.615$\times 10^{2}$	3.701$\times 10^{3}$	5.099$\times 10^{2}$	5.198$\times 10^{2}$	3.617$\times 10^{2}$
	SD	1.401$\times 10^{1}$	9.901	1.815	1.099$\times 10^{2}$	6.603	1.789$\times 10^{1}$	9.701 × 10^−1^
	Sign	=	=	=	+	=	=	
F28	Mean	4.501$\times 10^{2}$	3.312$\times 10^{2}$	3.601$\times 10^{2}$	3.214$\times 10^{3}$	2.901$\times 10^{2}$	2.888$\times 10^{2}$	8.498$\times 10^{1}$
	SD	6.501$\times 10^{1}$	3.919$\times 10^{1}$	5.097$\times 10^{1}$	7.501$\times 10^{1}$	3.883$\times 10^{1}$	5.803$\times 10^{1}$	3.206 × 10^1^
	Sign	+	+	+	+	+	+	
F29	Mean	5.099$\times 10^{2}$	8.412$\times 10^{2}$	6.504$\times 10^{2}$	3.811$\times 10^{3}$	4.415$\times 10^{2}$	3.901$\times 10^{2}$	2.640 × 10^2^
	SD	1.812$\times 10^{2}$	1.301$\times 10^{2}$	6.601$\times 10^{1}$	1.402$\times 10^{2}$	7.096	4.111$\times 10^{1}$	1.222 × 10^1^
	Sign	+	+	+	+	+	+	
F30	Mean	1.111$\times 10^{4}$	2.401$\times 10^{3}$	2.719$\times 10^{3}$	9.828$\times 10^{3}$	1.502$\times 10^{3}$	8.828$\times 10^{2}$	7.826 × 10^2^
	SD	5.801$\times 10^{3}$	5.242$\times 10^{2}$	9.401$\times 10^{2}$	3.615$\times 10^{3}$	4.299$\times 10^{3}$	9.099$\times 10^{2}$	2.606 × 10^2^
	Sign	=	$-$	$-$	=	$-$	$-$

**Table 5:** Wilcoxon signed-rank and friedman rank test based on mean error of CEC 2017 (D = 50).

Sign	COMFO	Vs.	BWM_HS	CVnew	SGSADE	HGSO	LSHADE-cnEpSin	LSHADE-SPACMA
+/=/−			27/00/02	22/02/05	26/00/03	28/00/01	17/04/08	18/03/08
Statistical rank		BWM_HS	CVnew	SGSADE	HGSO	LSHADE -cnEpSin	LSHADE -SPACMA	COMFO
Friedman rank		5.497	4.683	5.105	6.996	2.782	2.121	1.409
Overall rank		6	4	5	7	3	2	1

**Table 6:** Summaries of different case studies under consideration.

Case	Without renewable	With renewable	Considered objective	Constraints	Test system
1	$\checkmark$		Overall cost reduction with valve point effects		
2	$\checkmark$		Emission minimization	Equality and inequality	4-Hydro 3-Thermal
3		$\checkmark$	Overall cost reduction with valve point effects		
4		$\checkmark$	Emission minimization	Equality and inequality	4-Hydro 3-Thermal 1-Wind 1-Solar 1-Tidal 1-EV

### HTS systems

Using the moth flame optimization algorithm (MFO), in this simulation study two different hybrid test systems (HTS) *i*.*e*., one with and one without renewable energy sources, were examined in this simulation study. In addition, electric vehicles (EVs) and tidal energy were integrated with renewable sources to maximize the advantages of a virtual power plant. Brief descriptions of the test systems under study are made in Table 6. The systems data of the proposed research work are given in Tables A1–A13. The suggested MFO algorithm was contrasted with alternative optimization methods in order to evaluate its efficacy, including teaching learning based optimization (TLBO)^45^, clonal selection algorithm (CSA)^48^, differential evolution (DE)^49^, and improved particle swarm optimization (IPSO)^52^. The simulations were conducted using MATLAB 7.8 on a system equipped with a recent generation Intel Core i5 CPU running at 2.5 GHz and 4 GB of RAM. The results for both test systems were documented, detailing the lowest, average, and highest generation costs, as well as computation times. Each test system was performed for 100 iterations with a population size of 50. The MFO algorithm showed optimal performance with a jump rate of 0.3. Test System 1 consisted of four hydroelectric and three thermal power plants, focusing on minimizing generation costs while ensuring reliability and stability. In contrast, Test System 2 featured a more diverse mix of energy sources: four hydroelectric units, three thermal units, one wind unit, one solar unit, one tidal unit, and one EV. This integration aimed not only to reduce costs but also to enhance the sustainability and resilience of the power system. Both test systems were rigorously evaluated using the MFO algorithm, with outcomes compared to those from TLBO, CSA, DE, and IPSO. The comparison highlighted performance in terms of generation costs, computational efficiency, and effectiveness in managing renewable energy sources and their uncertainty. The detailed results demonstrated that the MFO algorithm outperformed the other methods, achieving lower generation costs and improved computational efficiency. These findings highlight the potential of MFO in effectively managing hybrid power systems, particularly those with a high proportion of renewable energy sources and advanced technologies like EVs and tidal power.

Moreover, COMFO has been used on both test systems. In contrast to HTS systems that rely on non-renewable energy, the simulation findings show that using renewable sources reduces generation costs.

**Table 7:** Thermal power generation of each unit for test system-I.

Hour	Thermal power plant (without renewable energy)		
	Plant1	Plant2	Plant3
1	30.4973	210.2592	229.5196
2	175	206.8581	139.7598
3	175	124.9438	139.7545
4	38.6093	124.9184	229.5196
5	33.2099	124.9149	229.5196
6	175	184.9776	139.7597
7	175	214.1778	139.7619
8	28.0166	213.6608	319.2794
9	175	216.967	229.5273
10	175	209.5819	229.5194
11	175	129.216	319.2793
12	175	280.0915	229.5196
13	175	224.9497	229.5196
14	102.6847	124.9079	318.7135
15	175	209.8205	139.7127
16	106.7563	228.6361	229.5196
17	98.5909	209.8133	229.504
18	175	207.445	229.5196
19	112.1642	215.022	229.5196
20	20.911	285.6258	229.5194
21	175	124.9043	138.831
22	33.7233	127.1845	229.5196
23	20.1978	127.6663	229.5196
24	102.6748	124.9079	139.7598

**Table 8:** Hydro power plant generation and discharge of each unit for test system-I.

Hydro power plant (without renewable energy)							
Generation (MW)				Discharge (×10^4^ m^3^)			
Plant1	Plant2	Plant3	Plant4	Plant1	Plant2	Plant3	Plant4
78.897	50.164	21.6346	129.0269	0.855	0.6	2.344	0.6
71.1537	51.296	10.1869	125.7437	0.727	0.6	2.416	0.6
72.1469	52.934	13.5952	121.6253	0.7391	0.6	2.296	0.6
67.3884	54.5	19.24	115.8221	0.672	0.6	2.156	0.6
65.9078	55.504	28.8517	132.0915	0.654	0.6	1.941	0.6
60.4452	55.994	36.7302	147.0931	0.582	0.6	1.73	0.6
86.594	63.8744	38.8545	231.7413	0.992	0.7228	1.646	1.219
86.1952	66.0102	37.8707	258.9692	0.99	0.761	1.643	1.429
86.3171	68.1556	36.0665	277.968	0.992	0.7952	1.703	1.61
77.2591	67.5065	35.2959	285.8382	0.8123	0.774	1.737	1.705
87.6747	71.7037	35.9696	281.1584	0.987	0.838	1.732	1.646
85.9891	64.6333	33.8516	280.9147	0.951	0.7179	1.795	1.643
87.2879	73.6041	33.9562	285.6831	0.969	0.8698	1.83	1.703
87.2055	74.8421	33.3614	288.2862	0.955	0.891	1.869	1.737
85.5299	77.0498	34.9773	287.9079	0.9166	0.9352	1.843	1.732
86.1193	76.1657	40.2437	292.5612	0.924	0.932	1.725	1.795
86.066	77.0497	44.7193	304.2573	0.924	0.98	1.614	2
88.767	76.1007	47.139	296.0246	0.9824	1.011	1.58	1.8689
84.836	77.0939	48.7902	302.576	0.9192	1.089	1.9988	1.9988
82.2878	78.0024	53.89	299.7646	0.8875	1.1786	2	2
54.7097	67.5631	56.3146	292.6818	0.5079	0.942	1.9381	1.9381
54.372	68.6724	58.3511	288.1773	0.5	0.975	1.9244	1.9244
54.7547	70.8399	59.3073	287.7167	0.5	1.064	2	2
60.5977	70.8617	56.4812	244.7185	0.5611	1.1236	1.4418	1.4418

**Table 9:** Hourwise hydral volume of four hydro units for test system-II.

Hourwise hydral volume of four hydro units			
U-I	U-II	U-III	U-IV
1004240	820000	1619210	1168000
1002160	840000	1523920	1088450
954850	845500	1367800	968660
966470	871100	1434260	851650
966390	844190	1463170	903390
986390	807300	1467750	956590
977050	717300	1498270	1049950
968230	719590	1493730	992100
984920	736570	1496600	1059280
1018420	766570	1641600	1079310
1043710	759980	1611890	1068720
1093710	768530	1580710	1120440
1136080	788530	1556570	1183400
1206080	789700	1545830	1200140
1224950	811270	1419240	1251320
1219710	811310	1425960	1333410
1211630	759360	1508030	1386480
1231440	728220	1556000	1392230
1175150	723460	1633540	1599890
1152920	735570	1676810	1573960
1160510	736920	1729400	1535220
1153570	763230	1754970	1492060
1150940	732870	1685170	1406100
1200000	700000	1700000	1400000

**Table 10:** Hourwise hydro power discharge and generation for test system-II.

Hour	Hydral discharge of four units hourwise				Hour	Hydral generation of four units hourwise			
	U-I	U-II	U-III	U-IV		U-I	U-II	U-III	U-IV
1	0.9576	0.6	1.6179	0.6	1	84.1206	50.164	54.2397	129.0269
2	0.9208	0.6	1.7729	1.0355	2	82.1658	51.296	47.6972	173.3565
3	1.2731	0.845	2.9188	1.3579	3	93.3492	67.3632	0	189.7976
4	0.5838	0.644	1.0562	1.1701	4	58.7438	56.1055	51.803	159.1551
5	0.6008	1.0691	1.884	1.1005	5	60.0888	78.5445	41.9494	158.9735
6	0.5	1.0689	1.783	1.2409	6	52.2064	76.1758	45.5879	178.3381
7	0.8934	1.5	1.2396	1.9852	7	79.8442	82.3307	54.5692	240.1551
8	0.9882	0.6771	1.8145	1.6347	8	84.2765	49.2	45.4259	213.1412
9	0.8331	0.6302	2.0336	1.2122	9	76.6757	47.1848	36.8669	187.6994
10	0.765	0.6	1.1382	1.5827	10	73.3896	46.9829	57.065	220.5203
11	0.9471	0.9659	1.9073	1.3455	11	84.873	68.3175	45.8916	200.7043
12	0.5	0.7145	1.907	1.2973	12	54.0991	54.7858	44.9481	202.3769
13	0.6763	0.6	2.1885	1.404	13	69.5086	48.318	31.2181	218.7092
14	0.5	0.8883	1.8733	0.9708	14	55.044	66.3193	45.0824	177.7636
15	0.9113	0.6843	2.9567	1.3955	15	86.9637	55.4462	0	225.3639
16	1.0524	0.7996	1.2328	1.0861	16	94.5226	62.6724	52.7151	203.2594
17	0.9808	1.2195	1.1789	1.6578	17	90.717	78.6296	54.59	260.9377
18	0.6019	0.9114	1.457	1.8158	18	64.3491	63.4239	55.0306	272.5893
19	1.2629	0.7476	1.105	0.8801	19	101.5889	53.9064	56.5646	200.8146
20	0.8223	0.6789	1.4887	1.4921	20	80.2073	50.3715	57.5769	265.6162
21	0.6241	0.8865	1.8484	1.5663	21	65.6602	62.7271	51.1811	268.5191
22	0.8694	0.6369	1.5142	1.8886	22	83.2123	49.3352	58.9448	287.9857
23	0.9263	1.1036	2.101	1.9646	23	86.5249	72.4992	39.9441	283.1451
24	0.5094	1.1287	1.6076	1.5497	24	55.8999	71.0589	56.4075	253.888

**Table 11:** Thermal, wind, EV, tidal, solar power generation for test system-II.

Thermal, wind, electric vehicle, tidal and solar generation of various hours						
TUNIT 1	TUNIT 2	TUNIT 3	WIND	EV	TIDAL	SOLAR
102.6587	40	229.7902	0	60	0	0
36.3823	129.4953	148.4131	21.3752	60	29.8187	0
103.571	40	140.7445	16.2302	18.9444	30	0
105.0533	40	116.3755	13.5792	19.1845	30	0
174.175	40.0541	50	16.4085	19.1012	29.9072	6.6206
103.8262	125.6414	158.1555	2.4873	0.223	30	96.9442
20.2615	55.1386	307.5528	8.7474	20.1269	29.0622	336.6627
93.7508	121.7377	307.9745	0	0	0	83.9942
174.2787	116.8376	319.9178	0	0.4511	0.246	124.8377
103.6712	124.6808	192.0843	20.3195	59.9997	11.7017	1181.8218
104.2432	207.7711	231.0235	0	0	8.6812	380.9959
102.8966	300	76.4671	15.2054	19.2534	30	1388.1904
103.0709	40	408.5411	0	0	4.8596	282.4631
174.6324	40	227.5914	4.3745	0	0	191.3539
105.9578	123.0495	198.8366	0	0	6.0375	273.8796
102.9698	124.4885	238.5461	18.1054	19.5951	30	793.0426
102.3447	52.2219	229.3298	0	60	0	540.0214
102.3329	249.7028	229.6617	0	19.6263	22.8446	231.2373
21.0877	295.0791	241.2665	0	60	30	80.2821
147.9466	124.7676	233.514	0	60	30	0
100.3001	208.4065	133.5558	0	19.6502	0	0
21.3625	209.6706	59.4889	0	60	30	0
106.9062	116.4441	52.4614	3.8568	60	28.2182	0
102.473	121.4427	50	0	58.8299	30	0

**Table 12:** Hourwise solar panel on/off status.

Solar panel on of status of various solar panel hourwise												
PANEL 1	PANEL 2	PANEL 3	PANEL 4	PANEL 5	PANEL 6	PANEL 7	PANEL 8	PANEL 9	PANEL 10	PANEL 11	PANEL 12	PANEL 13
1	1	0	1	0	0	0	0	0	1	1	0	0
1	1	0	0	1	1	1	0	1	0	0	1	1
1	0	1	0	1	0	0	0	1	0	0	1	0
0	1	1	1	0	1	1	0	0	1	1	1	1
0	0	0	0	1	0	0	0	1	1	0	0	1
0	0	1	1	1	1	1	1	0	0	0	1	1
1	0	0	0	0	1	1	1	0	1	1	0	0
1	1	1	0	1	0	0	1	0	0	0	0	1
0	0	0	1	1	0	1	1	1	1	0	0	1
0	1	0	1	1	1	0	0	0	1	0	1	1
0	0	0	1	0	1	0	1	1	0	0	0	0
1	0	1	1	0	0	0	1	0	1	0	1	1
1	1	0	0	0	1	1	0	0	0	1	1	0
1	1	1	1	0	0	0	0	1	1	1	1	1
1	0	1	0	1	1	1	1	1	0	0	1	1
0	0	0	1	0	1	0	1	0	0	1	1	0
0	0	0	1	1	1	1	1	1	0	1	1	1
0	1	0	1	1	0	1	0	1	0	0	1	0
1	1	1	0	0	0	1	1	0	1	1	1	0
1	0	1	0	1	0	1	0	1	0	0	0	1
1	0	0	0	0	1	1	0	1	1	1	0	0
1	1	1	0	0	1	1	0	1	1	1	1	0
0	1	1	1	1	0	0	1	1	1	1	0	1
0	0	1	1	1	0	1	1	1	1	0	0	0

**Table 13:** Hourwise cost of thermal, wind, tidal, EV and solar for test system-II.

Hour	Thermal cost hourwise ($/day)	Wind cost hourwise ($/day)	Tidal cost hourwise ($/day)	Tidal cost hourwise ($/day)	Solar cost hourwise ($/day)
1	1292.3094	21.3021	3.1258	0.0001	0
2	1305.9817	141.8371	6.4851	0.0001	0
3	1068.6457	92.5918	6.5641	21.5518	0
4	1160.1943	71.2755	6.5641	18.9397	0
5	1102.6768	94.133	6.5236	19.5124	1.5293
6	1446.1767	21.8436	6.5641	0.0001	22.9941
7	1515.8626	41.0903	6.1632	43.745	78.6692
8	1847.8741	21.3021	3.1258	0	21.1298
9	2066.2435	21.3021	3.0752	0.0006	33.3258
10	1601.2442	130.9814	2.1616	0.0001	267.3321
11	1762.5064	21.3021	2.1281	0	93.8761
12	1786.3135	83.9895	6.5641	18.776	320.2936
13	1843.8693	21.3021	2.3674	0	70.2063
14	1561.3305	25.0462	3.1258	0	49.8317
15	1628.6435	21.3021	2.2601	0	68.6962
16	1592.3474	109.4294	6.5641	17.0874	183.7334
17	1399.6558	21.3021	3.1258	0.0001	123.7457
18	2018.6048	21.3021	3.9841	18.2931	53.5333
19	1876.7366	21.3021	6.5641	0.0001	18.3186
20	1822.9531	21.3021	6.5641	0.0001	0
21	1528.8466	21.3021	3.1258	19.2502	0
22	1157.9735	21.3021	6.5641	0.0001	0
23	1141.9263	23.9345	5.8186	0.0001	0
24	1063.1391	21.3021	6.5641	0.0007	0
Cost ($/day)	36592.0554	1113.0796	115.6729	177.1578	1407.2152
Total cost ($/day)			39405.18

**Table 14:** Individual cost of each generating unit for test system-II.

Individual cost of each generating unit	
Thermal cost hourwise ($/day)	36592.0554
Wind cost hourwise ($/day)	1113.0796
Tidal cost hourwise ($/day)	115.6729
EV cost hourwise ($/day)	177.1578
Solar cost hourwise ($/day)	1407.2152
Total cost ($/day)	39405.1809

**Table 15:** Statistical comparison for cost optimization for both systems.

Algorithms	Best fuel	Average fuel	Worst fuel	Computational	Function
	cost ($/day)	cost ($/day)	cost ($/day)	Time (Sec)	Evaluation
COMFO (Test System-II)	39405.1809	39468.42	39602.85	17.24	3483
OMFO (Test System-II)	39506.38	39565.06	39679.88	18.41	6124
MFO (Test System-II)	39658.11	39698.02	40018.14	22.16	3566
COMFO (Test System-I)	41692.20	41705.64	41891.24	21.06	3129
OMFO (Test System-I)	41781.02	41935.06	41951.57	22.08	5327
MFO (Test System-I)	41789.94	41829.48	41991.82	22.08	3306
CSA^48^	42440.57	NA	NA	NA	NA
DE^49^	44526.10	NA	NA	NA	NA
TLBO^45^	42385.88	42441.36	42407.23	NA	NA
PSO^52^	44740	NA	NA	NA NA	NA
QTLBO^45^	42187.49	42202.75	42193.46	NA	NA

**Table 16:** Statistical comparison for emission minimization of both systems.

Algorithms	Best emission	Average emission	Worst emission	Computational	Function
	(lb/day)	(lb/day)	(lb/day)	Time (Sec)	Evaluation
COMFO (Test System-II)	7551.38	7559.12	7572.73	16.65	3382
OMFO (Test System-II)	7558.26	7577.86	7593.11	29.01	5145
MFO (Test System-II)	7562.44	7580.05	7597.33	20.42	3507
COMFO (Test System-I)	15838.24	15842.39	15857.36	15.96	3222
OMFO (Test System-I)	15842.17	15850.55	15859.99	27.22	4826
MFO (Test System-I)	15849.43	15861.72	15873.50	19.17	3218
PSO^52^	16 928.00	NA	NA	NA	NA
Fuzzy EP^53^	16554.00	NA	NA	NA	NA
IGA^54^	17659.00	NA	NA	NA	NA
DE^55^	18257.00	NA	NA	NA	NA

#### Test system 1

In this test system, seven different units are indicated in Test system 1, consisting of a combination of four hydroelectric and three thermal power plants. A new optimization method called moth-flame optimization (MFO) is used to examine the performance of Test system 1. The MFO method is used to search for the best global solutions. The input parameter of the thermal power plant is exerted from^8^. Reservoir inflows, volume restrictions, maximum/minimum limits, and generation coefficients are among the cost coefficients taken from^8^. The moth flame optimization (MFO) algorithm was evaluated against several other optimization methods to minimize generation costs. MFO achieved the lowest cost at $41,526.37 per day, outperforming other techniques such as clonal selection algorithm (CSA) with $42,440.57, differential evolution (DE) at $44,526.10, and teaching learning-based optimization (TLBO) at $42,385.88. Additional comparisons with particle swarm optimization (PSO), modified differential evolution (MDE), and quantum-inspired TLBO (QTLBO) also confirmed the superiority of MFO. The results, including hour-wise generation data and statistical analysis, demonstrate the effectiveness of MFO in optimizing hybrid power systems for cost efficiency and reliability. The thermal power plant’s input parameter is derived from^8^. Based on the discussion above, it can be concluded that the newly developed metaheuristic technique. is superior to other currently utilized ways due to its effectiveness in lowering generating costs and computation time. Table 7 represents the thermal power generation of each unit. Hydro power plant generation and discharge of each unit is shown in Table 8. Its durability and resilience are further demonstrated by the near proximity of the lowest, mean, and maximum generating costs that were obtained. Thermal power generation of each unit is displayed in Fig. 8.

#### Test system-2

The effectiveness of the proposed MFO algorithm is further validated by applying it to a more complex system, known as Test system 2. This system builds on Test system 1 by adding non-linear elements such as wind, solar, tidal energy, and electric vehicles (EVs), thereby increasing its complexity. The input data for the hydro and thermal power plants remain the same as in the earlier test, while wind system data is sourced from^56^ and solar and EV inputs are taken from^47^. Tidal system data comes from^9^. Test system 2 consists of four hydro, three thermal, one wind, one solar, one tidal, and one EV unit. By integrating these renewable sources and EVs, the system aims to further reduce generation costs while handling the additional complexity of the expanded unit mix. units are present. Table 9 lists the hydro power volume of various units for each hour using the suggested methodologies. Hydro power generation and hydro power discharge are listed in Table 10. Hydro power generation of each unit has been presented in Fig. 9. The volume of each hydro power unit has been displayed in Fig. 10. Thermal, wind, EV, tidal, solar power generation for test system-II are displayed in Table 11.

In a similar way, Table 12 lists the status of the solar panels. Here, a total 13 PV panels are used. The hourly costs of thermal, electric vehicle, tidal, wind, and solar power generation are shown in the table in Table 13. The individual cost of each generator unit is listed in Table 14. The comparison of statistical analysis obtained by different algorithms for Test system-I and Test system-II is shown in Table 15. Thus, Table 15 indicates that the lowest generating cost while employing MFO is 39405.1809 $/day for solar, EV, tidal and wind integrated system, which are quite less with respect to test system 1. The convergence graph illustrates how the resulting cost ($/day) varies with the number of iterations of the recommended technique. It is hypothesized that MFO starts to converge based on the convergence graph of the renewable energy linked system *i*.*e*. described that at iteration 22 with a minimum cost of 39405.18 $/day, and for the without renewable energy system MFO begins to converge at iteration 26 with a cost of 41526.3689 ($/day). Convergence curve has been displayed in Fig. 11. Generation of thermal, wind, solar, EV, tidal have been displayed in Fig. 12. The convergence graph illustrates the relationship between the generated cost (in $/day) and the number of iterations for the proposed Moth Flame Optimization (MFO) method. It indicates that the MFO algorithm begins to converge at around 22 iterations, achieving a minimum cost of $39,405.18. Fig. 13. displays the thermal power generation for each unit in Test system 2. Simulation results reveal that the generation cost for Test system 2, using the MFO algorithm, significantly outperforms that of Test system 1 in addressing the realistic non-linear problem. Additionally, the integration of renewable energy sources (RESs) with the hydro-thermal system contributes to a reduction in fuel consumption by thermal units, ultimately lowering overall generation costs compared to systems without RESs. The convergence rate of the algorithm is enhanced by advanced metaheuristic methods. Fig. 14 present the hour-wise generation costs for wind, tidal, solar and EVs, as well as the status of solar panels for Test System 2 is shown in Fig. 15 respectively. Microgrid stabilization is a crucial part of modern electrical systems to ensure a consistent and dependable supply of electricity. A single renewable energy source increases unpredictability as microgrid stability is weakened by varying needs. However, it has been demonstrated that the microgrid should be easily stabilized by utilizing a range of energy sources to balance supply and demand. To transition to a sustainable energy system, increase microgrid reliability, and prevent blackouts, this process is very much needful.

### Emission minimization (Test system-1 and 2)

The effectiveness of the proposed MFO algorithm is further validated by applying it individually for minimizing the emission for both test system-1 (*i*.*e*. without renewable) and test system-2 (*i*.*e*. with renewable). The input data for the hydro, thermal, solar, tidal and EV remain the same as in the earlier cases. By integrating these renewable sources and EVs, the system aims to further reduce emision while handling the additional complexity. Comparison of statistical analysis for emission minimization obtained by different algorithms is listed in Table 16. Table 15 indicates that the lowest emission while employing COMFO, OMFO and MFO are 7551.38 lb/day, 7558.26 lb/day and 7562.44 lb/day, respectively. Similarly for solar, EV, tidal and wind integrated system the emissions are quite less (*i*.*e*. 16306.94 lb/day using COMFO, 16309.63 lb/day using OMFO and 16317.92 using MFO) with respect to emission minimization without renewable energy. Simulation results reveal that the emission for both systems using the COMFO algorithm, significantly outperforms other approaches. Additionally, the integration of renewable energy sources (RESs) with the hydro-thermal system contributes to a reduction in emission by thermal units, ultimately lowering overall generation emission compared to systems without RESs. .

## Conclusions

The integration of renewable energy sources (RESs) with traditional thermal units presents a pivotal solution for achieving sustainable and resilient energy systems. In this study, we focused on optimizing the scheduling of wind-solar-electric vehicle (EV)-based hydro-thermal generation systems (WSEHTGS) to enhance economic efficiency and environmental sustainability. Leveraging the Moth Flame Optimization (MFO) algorithm, a robust meta-heuristic approach, we addressed the complex, nonlinear nature of WSEHTGS to minimize generation costs and mitigate environmental impacts, including greenhouse gas emissions. Through comprehensive simulations and comparative analyses against existing algorithms, our results demonstrate that MFO effectively balances the dynamic integration of renewable resources and EVs with thermal units. This optimization not only improves operational efficiency but also supports grid stability and reliability. By incorporating the dynamic characteristics of EVs and the intermittency of renewable sources into scheduling decisions, our approach contributes to advancing sustainable energy practices. Future Scope Looking ahead, several promising directions for future research emerge from this study: Our study underscores the potential of advanced meta-heuristic algorithms and hybrid optimization techniques, including machine learning and deep reinforcement learning, to further enhance the performance of WSEHTGS. These approaches could unlock new avenues for optimizing energy scheduling under uncertainty and variability. Integrating energy storage systems (ESSs), such as batteries and pumped hydro storage, into WSEHTGS frameworks offers significant potential to enhance grid stability and manage fluctuations in renewable generation. Future research could explore optimal coordination strategies between ESSs, EVs, and renewable resources Exploring the integration of demand response programs with WSEHTGS could leverage flexible load management strategies. Incentivizing EV owners to adjust charging patterns based on grid conditions can support peak load shaving and enhance grid reliability. Robustness analysis is crucial to assess the resilience of optimized schedules against uncertainties such as variable renewable generation and unexpected grid events. Developing strategies to mitigate risks and enhance system resilience will be critical for real-world deployment. Understanding the impact of policy and regulatory frameworks on the adoption of integrated renewable and thermal generation systems is essential. Evaluating incentives and regulations that promote sustainable energy practices will influence the scalability and effectiveness of optimized solutions. Developing real-time implementation frameworks for WSEHTGS in smart grids represents a significant opportunity. Exploring decentralized control strategies that empower local energy communities to participate actively in energy management could revolutionize grid operations. In conclusion, our research underscores the transformative potential of integrating renewable energy sources, electric vehicles, and advanced optimization techniques in achieving sustainable and resilient energy systems. Continued innovation and interdisciplinary collaboration will be essential in advancing these solutions towards a cleaner and more sustainable energy future.

## Supplementary Information

Supplementary Information.

## Data Availability

The datasets used during the current study are available from the corresponding author on reasonable request.

## Abbreviations

AHA: Artificial hummingbird algorithm
CBL: Chaotic based learning
COMFO: Chaotic oppositional moth flame optimization
CQOWOA: Chaotic quasi-oppositional whale optimization algorithm
CSMA: Chaotic slime mould algorithm
CWOA: Chaotic whale optimization algorithm
CO: Cheetah optimization
CRO: Chemical reaction optimization
CSA: Clonal selection algorithm
CCO: Crisscross optimization
DE: Differential evolution
EV: Electric vehicle
HSA: Harmony search algorithm
HTS: Hydro-thermal scheduling
LR: Lagrange relaxation method
LP: Linear programming
MIP: Mixed integer programming
MDE: Modified differential evolution
MFO: Moth-flame optimization
NP: Nonlinear programming
OGHO: Oppositional grasshopper optimization
PSO: Particle swarm optimization
PDF: Probability density function
POZ: Prohibited operating zones
QP: Quadratic programming
QPSO: Quantam behaved particle swarm optimization
QTLBO: Quantam inspired TLBO
QOCRO: Quasi-oppositional chemical reaction optimization
QOWOA: Quasi-oppositional whale optimization algorithm
RES: Renewable energy sources
SD: Standard deviations
SOC: State of charge
TLBO: Teaching learning based optimization
TES: Tidal energy system
V2G: Vehicle–to–grid
VPP: Virtual power plant
WOA: Whale optimization algorithm
WP: Wind power
