# Supplementary material for: Electric vehicle integrated tidal-solar-wind-hydro-thermal systems for strengthing the microgrid and environment sustainability
Source: Sci Rep. 2025 Apr 28;15:14888. doi: 10.1038/s41598-025-98594-9 (PMC12038037; doi:10.1038/s41598-025-98594-9)
Supplement: Supplementary file 1 — Supplementary Information. [file 41598_2025_98594_MOESM1_ESM.pdf]

Appendix

Table A1
Transmission loss coefficients on conventional system(*i.e.* without RESs)

B loss coefficients ($\times 10^{-7}$)						
49	14	15	18	19	20	19
14	45	16	16	18	18	18
15	16	39	14	16	16	16
18	16	14	40	15	16	15
19	18	16	15	42	19	42
20	18	16	16	19	44	19
19	18	16	15	42	19	46

Table A2
Transmission loss coefficients for 4-hydro, 3-thermal, 1-wind, 1-solar, 1-tidal, 1-EV system (*i.e.* with RESs)

B loss co-efficient for renewable based system											
$10^{-7}\times$	[49	14	15	15	16	17	17	18	19	20	19
	14	45	16	16	17	15	15	16	18	18	18
	15	16	39	13	12	12	14	14	16	16	16
	15	16	13	13	12	12	14	12	14	15	14
	16	17	12	12	13	12	13	13	15	16	15
	17	15	12	12	12	14	12	12	14	15	14
	17	15	14	14	13	12	17	16	16	18	16
	18	16	14	12	13	12	16	40	15	16	15
	19	18	16	14	15	14	16	15	42	19	42
	20	18	16	15	16	15	18	16	19	44	19
	19	18	16	14	15	14	16	15	42	19	46]

Table A3
Cost coefficients of the thermal generating system

Unit	P_i^{max} (MW)	P_i^{min} (MW)	a_i ($\$/h$)	b_i ($\$/MWh$)	c_i ($\$/(MW)^2h$)	d_i ($\$/h$)	e_i ($1/MW$)
1	175	20	100	2.45	0.0012	160	0.038
2	300	40	120	2.32	0.001	180	0.037
3	500	50	150	2.1	0.0015	200	0.035

Table A4
Emission coefficients of the thermal generating system

Unit	f_i (lb/h)	g_i (lb/MWh)	h_i $b/(MW)^2h)$	η_i (lb/h)	δ_i $(1/MW)$
1	60	-1.355	0.0105	0.4968	0.01925
2	45	-0.6	0.008	0.486	0.01694
3	30	-0.555	0.012	0.5035	0.01478

Table A5
Hydro power generation coefficients

Unit	λ_1	λ_2	λ_3	λ_4	λ_5	λ_6
1	-0.0042	-0.42	0.03	0.9	10	-50
2	-0.004	-0.3	0.015	1.14	9.5	-70
3	-0.0016	-0.3	0.014	0.55	5.5	-40
4	-0.003	-0.31	0.027	1.44	14	-90

Table A6
Hydro power reservoir inflows $(\times 10^4 m^3)$

Hour	I	II	III	IV	Hour	I	II	III	IV	Hour	I	II	III	IV
1	10	8	8.1	2.8	9	10	8	1	0	17	9	7	2	0
2	9	8	8.2	2.4	10	11	9	1	0	18	8	6	2	0
3	8	9	4	1.6	11	12	9	1	0	19	7	7	1	0
4	7	9	2	0	12	10	8	2	0	20	6	8	1	0
5	6	8	3	0	13	11	8	4	0	21	7	9	2	0
6	7	7	4	0	14	12	9	3	0	22	8	9	2	0
7	8	6	3	0	15	11	9	3	0	23	9	8	1	0
8	9	7	2	0	16	10	8	2	0	24	10	8	0	0

Table A7
Reservoir storage capacity limits, plant discharge limits, reservoir end conditions $(*\ 10^4 m^3)$ and plant generation limit (MW)

Plant	$V_{\min}$	$V_{\max}$	V_{bn}	V_{end}	$Q_{\min}$	$Q_{\max}$	$P_{hd,\min}$	$P_{hd,\max}$
1	80	150	100	120	5	15	0	500
2	60	120	80	70	6	15	0	500
3	100	240	170	170	10	30	0	500
4	70	160	120	140	0	20	0	500

Table A8
Wind mills data

Items	Plant-1	Items	Plant-2	Items	Plant-1	Items	Plant-2
No of WG	30	No of WG	20	v_{co}	25	v_{co}	25
m	4.6024	m	4.4363	$k_{o,wi}$	30	$k_{o,wi}$	20
n	1.8862	n	1.7128	$k_{u,wi}$	5	$k_{u,wi}$	5
v_{ci}	4	v_{ci}	3	l_{wi}	0	l_{wi}	0
v_r	16	v_r	13	wi_r	3	wi_r	3

Table A9
Power ratings and per unit rates of solar plants

Plant	$P^{*}_{so,rated}$ (MW)	Unit rate ($/kWh)	Plant	$P^{*}_{so,rated}$ (MW)	Unit rate ($/kWh)
1	20	0.22	8	40	0.27
2	25	0.23	9	40	0.27
3	25	0.23	10	40	0.275
4	30	0.24	11	40	0.28
5	30	0.24	12	40	0.28
6	35	0.25	13	40	0.28
7	35	0.26			

Table A10
Solar radiation, power demand and temperature

Time	Global solar radiation (W/m^2)	Power demand (MW)	Temperature in Celsius	Time	radiation (W/m^2)	demand (MW)	Temperature in Celsius
1:00	0	965	30	13:00	1013.5	1135	37
2:00	0	1142	29	14:00	848.2	1318	37
3:00	0	1177	28	15:00	726.7	1074	37
4:00	0	1198	28	16:00	654	1190	38
5:00	5.4	1153	28	17:00	392.9	1276	38
6:00	101	1136	-	18:00	215.1	1154	37
7:00	253.7	1138	29	19:00	38.5	1333	35
8:00	541.2	1060	31	20:00	0	1322	34
9:00	530.4	1155	33	21:00	0	1269	34
10:00	793.9	1244	34	22:00	0	1139	33
11:00	1078	1088	35	23:00	0	1202	32
12:00	1125.6	1240	36	24:00	0	1291	-

Table A11
Solar Unit Data & Power Limits

Unit	No. of Solar Panel	P_S^{min}	P_S^{max}	δ	ζ	r_c	R_{sd}	$C_{u,j}$	$C_{o,j}$
1	13	5	15	0.6	3	120	800	3	1.5

Table A12
Electric vehicle data & Power Limits

Unit	No. of EVs	P_E^{min}	P_E^{max}	δ	ζ	$C_{u,j}$	$C_{o,j}$
1	1	30	50	0.54	19.54	30	70

Table A13
Probability density function for renewable energy sources

Wind power generators plants				
Number	Rating Pwr (MW)	Weibull parameters	Cost parameters ($/MWh) KRw	KPw
20	60	α=9,β=2	3	1.5
**Solar power system**				
Rated power PPVr (MW)		Lognormal parameters		
40		ζ=6 ,ξ=0.6		
**Combined EV and tidal system**				
EV Rated power PEVr (MW)	Normal parameters	Tidal Rated power PTDLr (MW)	Gumbel parameters	
30	ρ=19.54 σ=0.54	10	Υ =220, τ =24.52	
